# Spirit of the Foreign Periodicals, &c.

**Published:** 1842-07-01

**Authors:** 


### Spirit of the Foreign Periodicals, &c.

---

### M. Dumas's Lectures on Organic Chemistry.

We learn from the pages of the French Medical Gazette, that this celebrated teacher has recently published the farewell Lecture, at the close of his course on Organic Chemistry, which he delivered last year at the Faculty of Medicine. From the high reputation of the author, and the increasing attention which is now paid to this most interesting branch of analytic science, it is sure to attract very general attention. One section of it is devoted to the investigation of what he calls the "chemical statistics of organized beings," wherein he endeavours to determine—first, the means of the nutrition and growth of animals and vegetables; and, secondly, the laws which maintain and perpetuate on the surface of the globe the relative existence of animals and plants.

In another section, the author discusses very elaborately the difficult questions of respiration, animal heat, and digestion, exposing, as he goes along, the fallacy of former theories, and propounding several novel views of his own.

All that we propose to do, is to select a few of the most interesting passages from the Lectures, and the comments of the French reviewer upon them.

*Source of the Component Materials of Vegetable and Animal Substances.*

"Whence is the matter of which plants and animals are composed? and whither does it go when death breaks the chains which bind their different parts together?"—these are the questions which M. *Dumas* propounds at the threshold of his inquiries, and which no doubt must often have suggested themselves to every reflecting mind.

Considered in the generality of living beings, the matter, which is incessantly organised and destroyed, will be found to proceed from, and to return to, the atmosphere surrounding us. It is this element which constitutes the bond of union between the two classes of living existences;—which is the grand laboratory whence plants and animals draw the elements of their mutual reparation; and which is also the immense reservoir that receives the products of the destruction of beings destined themselves to supply the materials required for the development of new existences.

Living plants are found to decompose the carbonic acid of the air for the purpose of assimilating its carbon to themselves; they decompose the water in the atmosphere or the earth, appropriating the hydrogen and liberating the oxygen; and from the atmosphere they derive the greater portion of the nitrogen or azote which enters into their composition.

On the other hand, animals are observed to be continually pouring forth a watery vapour into the surrounding air, in consequence of the re-action of its oxygen with the hydrogen of their bodies. The carbon combining with the oxygen generates carbonic acid; and nitrogen is exhaled, either directly in the gaseous state during expiration, or indirectly, in the form of ammonia, by the urinary excretion.

In this manner there is a constant reciprocal and compensating interchange of elements between the two classes of living beings: plants withdrawing from the air what animals communicate to it. In this chemical point of view, if the *former* constitute an apparatus, on a great scale, of reduction for appropriating the hydrogen of water, the carbon of carbonic acid and the azote of ammonia, the *latter*, in their turn, constitute an apparatus of combustion, in which carbon and hydrogen are continually burned, and from which azote is continually exhaled.

The atmospheric air may therefore be regarded as the important bond of union which holds together the two great classes of organised existences. Matter descends from it into the bodies of plants; it penetrates subsequently into those of animals; and ultimately returns to its primitive source, when the latter have fulfilled the purposes for which they were called into life.

The water and carbon, which are incorporated with the framework of plants, are not long of producing, under the wonderful but mysterious operation of solar light, all the organic compounds which constitute the different vegetable tissues. We only need study the composition of the constituents furnished by their analysis to be convinced that the various substances of gum, sugar, starch, &c. may be formed by the immediate combination of these elements; viz. water and charcoal, water, charcoal and azote or ammonia.

It is thus in the vegetable world, according to the opinion of M. *Dumas*, that the complex products of organisation are first prepared, and that the great primary laboratory of life is seated. From vegetables the materials pass into the bodies of herbivorous animals, and in these they become assimilated, and prepared to become the food of other tribes of animals. The fibrine, albumen, and caseum—which are generally regarded as the essential products of animal elaboration—exist already formed in plants; for in their constitution and in their properties, these components are strikingly analogous with the lignine, starch, and dextrine of vegetable substances..............

*Generation and Influence of Ammonia.*

Ammonia is the principal product of the urinary secretion. The azote, which is necessary for the development of vegetable substances, is in this form supplied either to the atmosphere or to the ground. But the organs, which constitute the reservoir and the excretory canal of the urine, would be inevitably injured by the direct contact of ammonia or even of its carbonate. Hence it is that it is not ammonia or its carbonate that is discharged in the urine, but another compound of hydrogen and nitrogen—viz. urea. Now, what is the chemical composition of this substance? It is found to be nothing else but carbonate of ammonia, deprived of two molecules of water. Thus modified, the carbonate is altogether inactive when applied to a living animal membrane; and then it can pass along the urinary apparatus without giving rise to any irritation. But when exposed to the air, it quickly undergoes a genuine fermentation, which restores to it its two molecules of water; and then the carbonate of ammonia, the formation of which is the final object of the urinary secretion, is found to be distinctly developed.

This salt, the carbonate of ammonia, is found elsewhere in certain physical conditions, which seem to indicate a remarkable foresight, so to speak, in the designs of nature. Although very soluble in water, it presents the singular and altogether exceptional property of completely abandoning its aqueous solutions, whenever they are freely exposed to the air. Being thus soluble and volatile at the same time, the salt is alternately exhaled into the atmosphere, and redissolved by the rain. In this way it can travel about from place to place, from the air to the earth, and back again from the earth to the air, until, after being elaborated in the interior of living plants, it is again converted into organic matter. If, instead of being volatile, it had been of a fixed nature, the result would inevitably have been that it would have remained attached to those parts of the soil, where it had been developed, and the general mass of vegetables could not have entered into those ready relations with it, which now exist throughout the world.

*Action of Plants on Carbonic Acid, &c.*

It is generally said that plants evolve carbonic acid during the night. M. *Dumas* is of a different opinion; he thinks that they only draw by their roots the carbonic acid contained in the soil, and that this gas incessantly traverses their tissues, to be finally exhaled into the atmosphere. During the day, the solar light induces the fixation of the carbon; but if the plant grows in the shade or during the night, no foreign force intervening, the carbonic acid escapes without any change.

At certain periods, a plant, or at least certain organs of it, acts the part of an animal, and becomes, like it, an apparatus of combustion for carbon and hydrogen, heat being evolved during the process. Let an embryo be developed, a flower be impregnated, or a grain begin to germ, and immediately a certain amount of caloric is generated, and carbonic acid and water are produced—in other words, vegetable substances exhibit all the characters of animal life.

An interesting subject for enquiry is to examine what is the part that the hydrogenous substances, which abound in so many plants, perform. Volatile oils seem to act as a defence against the attacks of insects; while the thick fixed oils, and the fatty matter which surrounds the grain, by undergoing a slow combustion during the process of germination, serve to produce the heat necessary for the development of the young plant. Again, the waxy matter, which varnishes the surface of many leaves and fruits, renders them impermeable alike to the exudation and imbibition of moisture. While we thus admit the importance of such hydrogenated compounds to vegetable life, it is not to be forgotten that they are neither so abundant nor so necessary as the numerous neutral products formed by the union of carbon and water...........

*Fermentative Processes in Living Bodies.*

M. *Dumas* has been led by the results of his numerous researches to give a remarkable extension to the influence of the process, so well known under the name of fermentation, and of which the conversion of saccharine matter into carbonic acid presents one of the best known examples, in the production of the phenomena of organic chemistry. A multitude of decompositions, to which animal and vegetable substances are subject, and which are apparently very dissimilar from each other, seem to be all owing to this process. As examples, we may cite the transformation of urea into carbonate of ammonia under the influence of the mucus derived from the bladder; that of the fecula of plants into dextrine and sugar; the acetification of alcoholic liquors; the putrid decomposition of organised substances; the viscous fermentation of sugar and the change of this substance into lactic acid; the simultaneous development of the oil of bitter almonds and of cyanhydric acid, that of the essence of black mustard; and, we may even add to the catalogue, the phenomena of nitrification. All these transformations, hitherto inexplicable, may according to this idea be accounted for, if there be only present a matter capable of playing the part that is performed by yeast in alcoholic fermentation. M. *Dumas,* it deserves to be mentioned, *regards all ferments as organised beings*...........

*Respiration, Animal Heat, and Digestion.*

The explanation, simple and beautiful as it certainly is, proposed by *Lavoisier* of the chemical process, which seems to constitute the phenomena of respiration in animals, has been for a long time known to be irreconcileable with a number of well-established facts. M. *Dumas* has suggested a different view of the question; frankly acknowledging that the idea was originally suggested to his mind by the writings of the German chemist M. *Mitscherlich.* He supposes that the lactate of soda, naturally held in solution by the blood, is converted by the action of the oxygen in the atmosphere into carbonate of soda. This salt, the carbonate, is then itself decomposed by the free lactic acid coming from the stomach, and the carbonic gas, being thus set at liberty, escapes into the air when the blood arrives at the exposed surface of the lungs. At the same time, the oxygen of the atmosphere, absorbed anew by the vessels during inspiration, continues to re-act upon the reproduced lactate of soda; and thus there is kept up a constant succession of these decompositions.

If this view of the question be correct, the process of sanguification would be very far from being so simple an act as *Lavoisier* and his followers have imagined. To appreciate M. *Dumas*' theory of respiration, it must be considered not isolated and apart from other functions of the body, but rather as constituting a sequence of his chemical doctrine of digestion.

Before explaining this doctrine we shall briefly allude to his opinions on that much disputed subject, the cause and origin of animal heat. We may state *in limine* that, according to his views, this important function is nothing more nor less than the result of a chemical action that takes place in the lungs—the combustion, or, in other words, the combination of carbon and hydrogen with the oxygen of the air. The amount of heat produced, he says, is exactly proportionate to the quantities of these elements consumed. Alluding to the doctrines of MM. *Dulong* and *Desportes*, who imagined that animals have the power of generating heat without any loss of matter expended, he remarks:—" These able physicians have supposed that if an animal be placed and kept for some time in a calorimeter with cold water, its temperature is little, if at all, abated. This however is well known in the present day not to be the case. It is this very cooling of the animal—not taken by these gentlemen into account,—which expresses in their tables the excess of heat that has been attributed by them and by most other physiologists to a peculiar power in the system of the animal, independent of respiration."

### *M. Dumas' Theory of Digestion.*

In estimating the novel views of our author on this subject, be it remembered that on all occasions he shews a strong desire to reduce under the dominion of the laws, which regulate dead matter, the greater number of the phenomena exhibited by living bodies. The great aim and end therefore of all his researches seem to be to render an exact account of all the varied re-actions which occur in the living economy. Under the influence of this idea—which, we must confess, is far from being satisfactory to our minds—M. *Dumas* strives to explain the various phenomena of digestion by the ordinary laws of chemical action. According to him, the transformation of the alimentary product into a substance, destined to repair the waste of the different organs, is nothing else but a simple function of absorption. The vegetable and animal matters which are used for food contain within themselves, and all ready formed, the immediate principles, which are first deposited in the digestive passages and subsequently pass into the vessels, without undergoing any important change. M. *Dumas* arranges the different articles used for food in three divisions; 1, substances which are capable of assimilation, such as fibrine, albumen, and caseum; 2, substances which are soluble, such as sugar, gum, and fecula; and 3, fatty or oleaginous substances. The *first* are, according to him, absorbed *en nature* by means of a chemical mechanism which has the effect of bringing the assimilable matter into a globular state, so as to permit it to enter and be conveyed along the minute absorbent vessels; the *second* are converted into lactic acid, which is destined—as we alluded to in the preceding page—to supply the essential elements of respiration; and the *last* are usually assimilated in their natural condition, but may be, as occasion requires, taken up by the organs, and consumed by combustion during the process of respiration.

It may be premature to impugn these views of M. *Dumas*, until a work, announced by him, on nutrition, makes its appearance. Still we must caution our readers against too readily adopting any theory of living action, which is based exclusively on experiments performed on dead matter. The animal body is indeed a laboratory, wherein numerous and most complex decompositions and recompositions are incessantly going on; but then all these processes are modified and controlled by that mysterious, but not the less indubitable, agency which we denominate life.

Much may however be expected from the researches of such men as M. *Dumas, Liebig,* &c. in revealing some, at least, of the steps or acts in these processes; and we may confidently predict that the beautiful science of physiology will henceforth derive by far its most important discoveries from the labours of the enlightened chemist. Away then with the detestable practice of vivisection; and welcome to the peaceful use of the microscope and the test tube.—*Gazette Medicale.*

---

### Another Blow to Broussaism in France.

Baron *Michel,* a physician of high standing in the French army, and one of the medical officers of the military hospital of *Gros Caillou* in Paris, has just published a work, entitled "Medical Statistics of this Hospital, followed by Theoretical and Practical Reflexions on Intermittent, Remittent, and Typhoid Fevers."

We select the following extract on the *Treatment of Typhoid Fever,* in illustration of the heading of this article.

"Having found by experience that bleeding and the use of purgatives effected very few cures, I have been led to believe that the phenomena of the disease are mainly attributable to nervous irritation, one effect of which is to suspend or modify all the secretions, and more especially those of the mucous membranes. To relieve this state of things, I have endeavoured to re-establish the functions of the skin—the state of which has such intimate connection with that of the mucous membranes—and at the same time to calm the nervous irritation by the use of appropriate soothing medicines. To effect these ends, my chief remedy has been the acetate of ammonia combined with laudanum.

As soon as a patient exhibits the symptoms which indicate the approach or invasion of typhoid fever, such as general prostration, dryness of the skin, thirst, parched tongue, &c. I administer frequent doses of the medicine in large quantities of mucilaginous water.

From the second or third day, when the disease is really curable, and when it is not associated with organic lesions, the tongue becomes more soft and moist, the skin begins to perspire, and the secretions of the mucous membranes to be re-established....... This mode of treatment has the very great advantage of abridging materially the length of the convalescence, and restoring the patient much more rapidly to health, than if he had been treated on other principles. When a patient's strength is much reduced by depletory remedies used for the cure of fever, it often requires three, four, or even six months before he is again fit for military service."

M. *Michel* appends some statistical tables, from which it appears that of 6561 patients admitted into Gros Caillou Hospital during 1838, 429 were affected with typhoid fever: of this number 90 died.

On the subject of intermittent fevers, the following extract will shew the utter inefficacy, nay rather the pernicious effects, of what has been so absurdly called the physiological method of treatment. It so happens that Baron *Michel's* work has somehow or other, and without his sanction, been accompanied with critical notes by M. *Cassimir Broussais,* who seems to think it only an act of filial duty to maintain the now waning doctrines of his father. In his comments upon the ætiology of agues, he tries to support the idea of this important class of fevers being dependent upon an inflammatory condition of the nervous centres by calling to his aid the opinions of the military physicians and surgeons who have of late years had such frequent and extensive opportunities of studying them in Algeria. Unhappily for him, however, the experience of these gentlemen is any thing but flattering to his views; for if there is one place more than another, in which the physiological doctrines have met with more decided reverses, even in the hands of their most devoted admirers, it is unquestionably in Africa.

"At Bona and at Algiers," says Baron *Michel*, "during the years 1832 and 1833, the disciples of this school saw nothing in the intermittent and malignant fevers of the countries but acute gastro-enteritis and gastro-cephalitis, and accordingly based their practice upon this view of the question. It was soon discovered that this was an utter fallacy, and that the only method of successful treatment was by administering large doses of quinine, with but little regard to the nature or severity of the symptoms present. So decisive were the effects, that the most ardent Broussaists speedily abandoned their antiphlogistic measures, and with gladness embraced the method which produced such pleasing results."

One of the chief surgeons of the army writes thus on the subject: "How many of us have to be thankful to M. *Maillott* for the method of treating the fevers of Bona which he introduced; for, before that was employed, it must be confessed that the results were in truth most unsatisfactory. The losses have been considerably less since we adopted the practice of exhibiting bark freely. My testimony on this subject cannot be suspected; for, believe me, it required no common evidence to induce me to abandon a doctrine in which I had previously so much confidence."—*Gazette Medicale.*

*Remarks.*—If there is one subject of practice on which French medical men are more at sea—without a compass too—than another, it is unquestionably that of the treatment of fevers. We cannot wonder at this, when we think of the mad enthusiasm with which any favourite doctrine is embraced and inevitably carried *à l'outrance*. *Broussais* has had his day, and a pretty long one it has been. And what has been the result of the dominion which he has exercised over the minds not only of his countrymen, but also of many in other countries? We hesitate not to say, a deplorable loss of human life, and a decided retrocession of medical science. It is only now that the French are beginning to find out that the *gastro-enterite* doctrines are, in a great measure at least, an utter and a most pernicious fallacy. To attribute all the complicated symptoms of fevers to a slight inflammation of the mucous membrane of the stomach or bowels appears to be so irrational that we wonder how any sane man could ever have been misled by it; and yet, year after year, flocks of medical students have issued from the French schools as thorougly impressed with this notion, as that pain is produced by irritating a nerve, or that a full dose of opium will induce sleep.

This state of things is passing away; and perhaps now the chief evil that we have to guard against is, that the doctrine of local irritation may be too much overlooked, while some other doctrine takes its place, and reigns paramount in its stead. Let it be remembered that there is a certain amount of truth, blended indeed with a vast deal of fallacy, in the tenets of the *Broussaian* creed; and therefore that the total abandonment may be nearly as hurtful as an exclusive and indiscriminate adoption of them.

Judging from the practice of Baron *Michel*—at least as it is given in the Gazette Medicale, for we have not seen the original work—there is a tendency among some of the French physicians to substitute a mode of treatment that is altogether empirical for one that is derived solely from theoretical speculation. To recommend the liquor ammoniæ acetatis in conjunction with small doses of opium in all cases of fever indiscriminately, would be little better than an open avowal of downright quackery.

How comes it that no mention is made of emetics—unquestionably among the most important of all remedies in the early stage of all fevers—or of purgatives, or of leeches, blisters, and so forth? It cannot surely be that these means are utterly powerless in the treatment of many epidemics of typhoid fever, or that they can be replaced by a single formula, and that formula composed of the spiritus mindereri and laudanum. We have so often expressed our opinions on the management of fevers that it is almost unnecessary to add anything more at present. We are friendly to the use of mild diaphoretics—of which, by the bye, the employment of the tepid bath, or tepid ablutions is certainly one of the best—but not to the exclusion of other important remedies.—*Rev.*

---

### On the Use and Abuse of Corsets.

A lively and intelligent correspondent of the Gazette Medicale has addressed two or three letters on this most important part of women's dress to Madame ———, and with the usual cleverness of a Frenchman on such matters, tries to gain the end he has in view—that of exposing the injurious effects of tight-lacing—by artfully blending witty satire and graceful flattery with the arguments and reasoning that he employs. It is only the last letter which we have seen: the former ones must somehow or other have escaped our notice. Here are a few specimens.

"Judging from the tone of your reply, Madam, you seem to be convinced yourself of the serious evils of the corset; but while you admit the truth of what I have been saying, you candidly confess that all my preaching will make but few proselytes among the ladies. I know it; but if even a few are gained, it is worth my trouble. In vain may medical men advise, warn, and demonstrate the truth of their precepts; the *beau sexe* will not the less continue to brace in their shoulders and chests, until they often become positively deformed. There is but one way of reaching the difficulty, and convincing the ladies of the absurdity and danger of the practice; and that is to shew that the use of the corset really spoils their shape, and takes away from their attractions. If we could but once make the dear creatures believe that it is the resource only of those who are somewhat deformed, and that the necessity of wearing a corset is a proof in itself of a want of natural grace on the part of the wearer, then indeed might we hope to gain our end. Should *fashion* ever pronounce these few words, *le corset vieillit et enlaidit*—by the bye, these two words are not easily translated by single synonyms—and if it should add, 'do you wish to be always lovely and graceful, ever armed for new conquests? have nothing to do with it;' from that moment its empire is gone, and we shall not have even a relic left of this barbarous and most foolish piece of armour.

The question therefore must be simply, 'does the corset embellish natural grace?'—since all considerations about health and comfort go, as a matter of course, for nothing in the solution of it. Now where is the *man* that will not allow that it really spoils the shape of a fine woman, and that does not prefer the beauty of Nature's form to all the sublime imaginings of the tailors and sempstresses of Paris?

But you will perhaps say, 'Doctor, are you not too bold to make yourself a judge of the toilette of the ladies?' Madam, I readily admit my utter incompetence; the art is far too difficult and complicated for me to understand it. To attempt to speak upon it would be to get myself laughed at by all the young coquettes, in whose eyes a doctor is only a dark dismal man, the object not unfrequently of mirth and ridicule."

"There is just as much barbarism in French women drawing in their waists and cribbing up their shoulders, as in the pinching of the feet by the Chinese, or the flattening of the head by some of the Indians. How a spider-waist should ever be regarded as a sign of beauty, puzzles us poor men mortals to understand. Where is the sculptor or painter that, for his own sake, would represent a woman exactly as she stands before him in all the unnatural restraint of modern dress? Whenever the natural expansion of every part is not permitted, and the natural freedom of movement is confined, there cannot be perfect grace and beauty. Only imagine a *Venus de Medecis* in a corset! the very idea is too absurd to be thought of; and yet there is not a woman in ten thousand but believes that she becomes more graceful and attractive, when once she is well tightened in with her stays."

"The following table of the various requirements in the composition of a perfect female beauty is taken from an old French work, entitled 'De la Louange et Beauté des Dames.'

| | | |
|---|---|---|
| Three things should be | white.... | the skin, the hands, the teeth. |
| | black.... | the eyes, the eyelashes, the eyebrows. |
| | red ..... | the lips, the cheeks, the nails. |
| | long .... | the body, the hair, the hands. |
| | short.... | the teeth, the ears, the feet. |
| | wide .... | the chest, the forehead, the space between the eyebrows. |
| | narrow ... | the mouth, the figure, the ankle. |
| | plump ... | the arm, the thigh, the calf of the leg. |
| | slender .. | the fingers, the lips, the hair. |
| | small .... | the breasts, the nose, and the head. |

Now, Madam, if there be any truth in this table, you see that a slender waist is only a small fractional part in the composition of perfect beauty; and yet it is to this fraction that most women attach so much importance—probably because they fancy that they can supply by art what has been refused by nature. In the *ensemble* of attractions enumerated above, there is not, and there cannot be, any single one that takes the pre-eminence over all the others. It is the life, the animated grace that gives the charm to each and to all of them; and how can that be when the restraint of art is substituted for the freedom of nature. *Ninon* very justly remarked that 'beauty without grace is a hook without a bait.' A woman can be really beautiful only in one way; but she may be pretty and graceful in a thousand different ways. If we are asked, what do you mean by grace as applied to a woman, we should say that it consists chiefly in her attitudes and general carriage. This is the secret of that inimitable *desinvolture,* peculiar to some women, and which is to be acquired only by the light and easy movements of the body. It is almost needless to say that perfect freedom can never be exercised, if the body is cabined, cribbed, confined by the stiff stays of the present day. A woman can then neither stoop, nor bend backwards, nor to a side without irksomeness and trouble. The pliancy and flexibility of the back, so essential to graceful movement of the body, become, as is well known, less and less as years advance, till at length in old age they are almost entirely lost. Are we not therefore quite correct in saying that the corset not only *enlaidit* but also *vicillit* the French figure?"

........ "If the most *spirituel* expression of the female character, next to the voice, is to be found in a graceful figure, may we not at once infer that, just in proportion as this expression exhibits the greatest amount of variety and of lights and shades, so will it be more lively and attractive. A witty man once said, 'There are some movements of the petticoat that deserve one of the *Monthyon* prizes:' this is quite true; but how comes it that so few women understand how to avail themselves of the advantages which they have in their own power. The truth is, that they imagine that all the art of attraction consists in holding themselves very erect, and in being well *corsées* and well *busquées.*"

"Without regular exercise in the open air, and perfect freedom of movement, the health—and without health how can there be perfect grace?—cannot possibly be preserved. Beauty, like other flowers, needs exposure to the air and the light of the sun. Now if you wish to have that bloom of spring-tide freshness, that rosy tint which no painter can imitate; if you desire that your walk should have grace and ease in its movements, remember that it is absolutely necessary that the blood should circulate without restraint or impediment, and for this end you must breathe freely, and walk and even run easily; in other words, that you must exercise your body and limbs regularly, actively, and with perfect freedom. The Duke of Saint-Simon very happily said, that the Duchess of Burgundy seemed to walk 'sur la pointe des fleurs.' You may be assured that she was not cased up in a huge heavy corset. Do you suppose that the dancing girls of India, so celebrated for the wonderful vivacity of their movements, wear such a machine? No, no; they have nothing but a light *corsage* provided with an elastic web, which supports without confining the body. You perhaps remember, Madam, that fine line, which *Carlo Maratti* inscribed below a picture of the Graces—*Senza di noi, ogni fatica è vana.* Now it is precisely by that exquisite beauty of form, which antiquity has given to them, that these celebrated figures charm every beholder. But who could recognise them as the eternal models of perfect beauty, if they were enveloped in the dress of modern times? Take even the example of some women who have shewn more than ordinary powers of fascination, and it will be found that they did not trust to the mere shape of their waists, but rather to a fine taste in the happy adjustment of their whole *parure,* so as to produce a harmony with their eyes, their figure, their complexion, and the general *ensemble* of their persons. No one understood this art better than the Empress *Josephine;* and it is well known that the corsets, that she wore, were very small and light."

"Would that women never lost sight of that most true and valuable maxim, that the dress ought never to be more youthful than the age of the party. Coquetry knows how to imitate every thing, even to innocence and modesty; but it requires the most delicate and refined taste to adjust nature and art so to produce an agreeable and harmonious whole. Many women are so alarmed at the idea of becoming lusty, that they will submit to the most severe tightening to prevent the supposed deformity. What a mistake! Most men rather like a woman, if not very young, to have a certain degree of *embonpoint;* and think you that it is youth alone, or the semblance of it, that is the only attraction which the fair have in the eyes of our sex? The experience of every day is against the idea; and every one could tell of examples of women, who had reached the *mezzo cammin di nostra vita,* retaining in the chains of their fascination men who had resisted all the allurements of mere youth and beauty. Hear what that gallant old knight, *Brantome,* says: 'Thus we often see many sorts of Winter fruits of last season equal those of Summer, and be as beautiful to look upon and as savoury to the taste.' Quite true, old Cavalier! It is not the girl alone, just escaped from her teens, and fantastically adorned in all the *graces* (!) of modern fashion that carries captive the eyes of every beholder. But most women have themselves to blame for the preference that is usually shewn to young beauties; not content with the charms which Nature has given them, they vainly strive to make up by artifice what they foolishly imagine to have been denied them."

---

### Notice of M. Vidal's Treatise on Surgery.

From the reviews in the French journals of M. *Vidal's* large work, entitled

*Traité de Pathologie Externe et de Medecine Operatoire,* in five volumes, published 1839—41, it appears that his countrymen are disposed to estimate its merits very highly, and to regard it as likely to occupy the place which has hitherto been so long held by Baron *Boyer's* system of surgery. We have not seen the work ourselves, but from various extracts we should be inclined to think well of it as a scientific and able performance. We shall select a few of these extracts that the English reader may judge for himself.

*Rashness of Modern Surgeons.*

"I believe with *John Bell* that we have too decided a taste for bold and hazardous enterprises, and consequently too great a distrust for the autocracy of Nature. It will be found that I lean to what may be called *le naturisme chirurgical;* but this is far from any bias on my part to a reaction which should propose a disarmament of surgery."

This rebuke ought not to be disregarded by surgeons; there is much truth in it. Many of the crude and hasty performances in operative surgery of late years have been anything but creditable to the profession. Witness the ignorant *furore* with which even so many respectable men have, almost indiscriminately, been cutting muscles and tendons across for the cure of squinting, stammering, spinal deformities, &c. &c. We are certainly not quite so bad as our brethren on the continent, who actually permitted, the other day, a charlatan to try his murderous proposal of rapid and forcible extension of long stiffened and even anchylosed joints, by means of a powerful mechanical apparatus! The Academy have at length, we are happy to observe, denounced the practice as altogether inadmissible.

*Surgical Diagnosis.*

"Let not the young practitioner ever attach too much importance to any single symptom, however valuable that may be, of a disease; for none is infallible. I would compare the symptoms of a disease to the threads composing a cord—which may represent the diagnosis;—each of the threads by itself is unable to support a weight which is readily borne by the entire cord. If I might carry out this comparison a little further, I would say that each thread is one degree of evidence, and that their union alone can constitute the certainty."

This is well and truly said; the comparison is very just, and happily illustrates the idea. The more experience we have, the more distrustful we become of any one symptom, or even one set of symptoms, taken apart; and, we may add, the less reliance have we in any one remedy or one mode of treatment. What pray is the cause, we might here ask, of the certain degree of re-action against the value of the stethoscope that has been manifesting itself during the last two or three years? Nothing more or less than the exaggerated importance ascribed to it, some time ago, as a means of diagnosis. It is a valuable instrument in the hands of the judicious physician; but its use should never supersede the ordinary means of diagnosis by what have been, absurdly enough, denominated the *rational* symptoms. Auscultation is, to use M. *Vidal's* illustration, one thread —a stout and good one we admit—in the cord; but it is not the cord itself; and if trusted to alone, will often prove fallacious. There are two methods of diagnosis, says our author; the method *à priori,* and the method of exclusion. To us it seems that these two methods are not so distinguishable as M. *Vidal* supposes: in fact, they are generally blended together. Take for example the case of a tumor in the groin; this at once suggests to the mind of the surgeon the idea of a hernia, and perhaps, on further examination, he finds that all the other symptoms confirm the accuracy of this idea. Now such is the diagnosis *à priori.* But this is not enough; the method by exclusion must now have its turn, and we must proceed to ascertain that the tumor is not an aneurism, a varix, a chronic abscess, &c. Thus the diagnosis by exclusion, or, what may be called, the differential method of diagnosis, serves to complete the diagnosis *à priori.*

*Operations en deux Temps.*

M. *Vidal,* in discussing this very interesting and important subject, criticises with much ability a passage in the last edition of *Sabatier's* work, where it is said that, "when an operation is commenced, the surgeon ought not to allow himself to be stopped by any obstacle." He remarks with great justice that, even in the best considered operations, something unforeseen will not unfrequently occur.

"Operations *en deux temps,*" says he, "have this advantage, that nature is not taken by surprise. In the first period, which is the least dangerous, the surgeon explores, so to speak, the ground on which he has to work; if he discovers, by the character which the wound exhibits, that the organism is in an unhealthy condition, the operation is adjourned, until the health is re-established so as to warrant the completion, or the second period, of the operation. The first period, moreover, draws towards the seat of the operation the re-action necessary for its success. Besides this consideration, there is in some cases considerable danger of removing all at once a disease which has existed for a length of time. Observe what occasionally happens after the excision of a large tumor—a general prostration of the vital energies from which the system may not recover. The organism seems as if it had lost its counterpoise; it has no longer that sort of ballast, so to speak, the sudden removal of which will probably make the vessel founder."

We are much pleased with the generous and truly Christian spirit evinced in the following remarks on operations:

"Sometimes," says M. *Vidal,* "it is a labouring man, the father of a family, who is about to undergo an operation which will disable him afterwards from providing for the wants of himself and his children. This is doubly distressing; and surely it is but the duty of the surgeon to comfort him at the time and promise to become his future benefactor and friend. How many noble actions might some great surgeons have done, if their souls had been commensurate with their talents and their fortune!"

A truly magnanimous sentiment; let the surgeons of the present day take it at once as a kind rebuke and as a most valuable lesson!

*Treatment of Gunshot Wounds.*

M. *Vidal* strongly recommends the application of leeches around gunshot wounds, and in the neighbourhood of contusions generally. "However," he adds, "it is necessary to bear in mind the distinction which I have pointed out between the two kinds of tumefaction which are apt to occur after such accidents. One is cold, indolent, and passive, and requires the employment not of any sedative or depressing applications, but rather of such as are warm and stimulating. Let it however be kept in mind that the effect of such remedies should always be attentively watched; for, when any symptoms of local reaction come on, we should at once discontinue their use, and resort without delay to those that are decidedly antiphlogistic."

*The Diet in Cases of Severe Wounds.*

Our author's opinions on this subject deserve the notice of the English, as well as of the French, surgeon. These are his words; "perhaps in France, we are somewhat too severe in the restriction of nutritious food, and keep our patients too long on a low diet. My own experience confirms the truth of the observation that purulent absorption goes on more readily in persons who are not sufficiently nourished during the healing of their wounds. The state of a wounded patient often resembles very strikingly that of a puerperal woman.

After the expulsion of the placenta, the internal surface of the uterus is in a condition very analogous with that of a large wound; and we all know that, as a general remark, lying-in women require an ample allowance of nutritious food. The English surgeons, who follow this plan after amputations, obtain perhaps more success than we can boast of in France."

*Torsion of Arteries.*

"Of late years some surgeons have endeavoured to substitute, in the treatment of hæmorrhage, torsion of the bleeding vessels for the use of the ligature. I have examined this question with much care, and must frankly confess that, in my opinion, the ligature is decidedly the preferable means; the application of it is simple, easy, not attended with pain, and not generally liable to be followed with unpleasant consequences. In the hospitals of Paris, the occurrence of hæmorrhage after a well-applied ligature is very rare indeed. The ligature is, in my opinion, to be preferred whenever we have to do with the lesion of an important artery."

The French reviewer of M. *Vidal's* work very judiciously remarks that the above opinion is rather too absolute and peremptory. Torsion of bleeding vessels will be found to be a very valuable means of arresting hæmorrhage in certain cases, where the application of a ligature may be exceedingly difficult or even impracticable. As we might anticipate, it is to arteries of the second and third order that torsion is chiefly applicable; for example, after excision of the mamma, amputation of the fore-arm, &c. In a recent case of strangulated hernia, in which we assisted M. *Velpeau*, the omentum was found, upon opening the sac, to be united by an old adhesion to its inner surface; when divided, its vessels bled profusely; torsion was employed, with the best effects. In such a case as this, the substitution of torsion for the use of a ligature is decidedly a great advantage gained, as no foreign substance is left in contact with the bowels; and it is unnecessary to say how liable these organs are to inflammation from the presence of any irritating matter. Like every other new remedy, torsion has been overrated by some and under-rated by other surgeons: it is the *juste milieu*—provided this be based upon rational grounds—that seems to be so difficult, and yet is so important, to hit.

*Remarks on Lithotomy.*

We all know how numerous have been the plans recommended at different times for the performance of this formidable operation. Many practised writers have experienced not a little difficulty in even classifying the various proposals that have been made, more especially in reference to the perineal operation, and we believe that there is scarcely one surgeon who, if examined on the question without previous preparation, could arrange his ideas on the subject so clearly as to be able to explain them satisfactorily to others. M. *Vidal* has done much to disentangle this confusion, and our readers will probably be pleased with the following very useful remarks on the classification which he recommends.

"The distinction of lithotomy into the *perineal*, the *hypogastric*, and the *rectal* methods is based on the consideration of the parts that are first divided in the performance of the operation. It is thus upon the *external* incision that most writers have founded the classifications which they have adopted. But this is certainly not the most important period of the operation; of much more consequence is the *internal* incision, that which is made upon some part of the urinary apparatus, the urethra, the prostate, or the bladder. According as the internal incision is limited to the urethra and the prostate, or involves at the same time part of the bladder itself, we have reason to expect a great difference in the results. As the consequences of the operation depend infinitely more on the mode and place of performing the *internal* than the *external* incision, it is surely more logical, and withal much more practical, to divide lithotomy into the *urethral*, the

*prostatic*, and the *vesical* methods, than into those which have usually been spoken of."

This classification will be found to facilitate very much the exposition of the very numerous methods which have been proposed for the performance of the operation in question; and it has this striking advantage over all others; that it directs the attention of the reader mainly to the essential point, viz.: the *internal* incision.

What has been called the *quadrilateral operation* is so truly the personal property, so to speak, of M. *Vidal* that we gladly avail ourselves of his own description of this method, which has attracted of late years no inconsiderable attention.

" I proposed this method in my inaugural thesis published in August 1828, my earliest attempts having been made three years earlier. It is one of the numerous applications of what has been called *debridement, multiple*,—a principle in operative surgery of which the advantages are now universally acknowledged. I felt convinced that the disciples of *Lecat*, who divided the prostate only partially but lacerated it freely, and those of *Cheselden*, who adopted the very opposite plan—that of dividing it freely and lacerating it very little—were alike at fault, but certainly in different degrees. The plan of *Lecat* was good, when the surgeon had to do with a small calculus; a small incision was then sufficient, and there was no occasion of enlarging it in any way. According to my view of the subject, the size of the calculus does not require an increase in the *extent*, but only in the *number* of the incisions; for the extent or depth ought to be in all cases nearly the same. Thus for the extraction of small calculi, one limited incision is sufficient (the unilateral operation); for those of a middle size, we should employ two incisions (the bilateral operation); and when the calculus is large, four small incisions should be made (the quadrilateral operation).

In my opinion, the mode of making the external incision is not of much consequence; it matters little whether this be parallel, oblique, perpendicular, straight, or curved; the great object of the surgeon should be that it is not too small. My practice may be briefly explained in these few words:—*one large external incision, and several small internal incisions.*

I prefer, on the whole the crucial external incision, as it was frequently performed by Dupuytren.

The two first incisions of the prostate are made upon its two oblique inferior radii. When the stone is of a moderate size, these two incisions will be found to be sufficient; but if the calculus be large, the two incisions unite into one, the edges of which will be stretched and lacerated in the efforts made to extract it. Under such circumstances, the surgeon should introduce his left fore-finger, provided with a long probe-pointed bistoury, having its cutting edge directed upwards, outwards, and to the left side (the left superior oblique radius) to the bottom of the wound, and afterwards direct it in the opposite line, viz. outwards, upwards and to the right side (the right superior oblique radius). If the surgeon wishes not to let go the calculus, he should entrust the forceps to his assistant, and this instrument will guide the bistoury, with which he is then to make the two upward incisions; these, instead of being prolonged towards the skin, (as is the case with the two inferior incions which are confounded with the wound of the perineum), should be limited to the substance of the prostate alone. In this manner the lithotomy becomes *quadrilateral within*, while, externally, it is only *bilateral;* the resistance to the exit of the calculus being greatest at the cervix of the bladder. We thus see that the number of the internal incisions should correspond with the size of the calculus, ever bearing in mind the danger of extending the incisions about the neck of the bladder in any one direction only. Once that a calculus has passed the opening in the prostate, it meets with tissues which readily yield to its passage outwards: thus the two inferior *debridements*, which come to be confounded with the outward crucial incision, afford ample room for the exit of the calculus from the perineum. It is not at the perineum that the calculus meets with any serious obstacles to its exit; it is during its passage from the bladder into that portion of it which corresponds with the membranous part of the urethra.

I foresaw that my proposal of the quadrilateral operation would lead to the contrivance of new instruments, and I alluded to this matter in my thesis in these words:—I hope that the profession will not expect from me an instrument with four blades, for the purpose of dividing the four radii of the prostate at the same time. *Chaussier* very aptly remarked, that nothing proves better the poverty of our art than the richness of its mechanical arsenals. Now this precaution has not prevented a very ingenious surgeon from conferring upon my method a superb four-bladed lithotome—which, however, very fortunately can never be made use of. One word will prove this. The quadrilateral section is required only when the calculus is of large size, and must consequently fill up a large part of the bladder. Conceive, then, to yourselves an instrument which, when introduced into this bladder, should expand into four blades, and the *deploiement* of which would require four times more space than actually exists! Moreover, in withdrawing such an instrument when expanded, an external quadrilateral incision would be made; and this I wish to avoid. The quadrilateral section, be it remembered, is made to replace the free section of the bladder in a single direction in cases where the stone is of a large size.

My operation was soon appreciated by many practical men; but, as I had not performed it on the living subject at the date of my proposal, its introduction into actual practice was slow.

First M. *Velpeau,* who is always one of the foremost in any surgical movement, and then M. *Guersent,* made the earliest trials of it in Paris; and MM. *Goyrand, Rolland,* and *Roux-Martin* of the provinces followed their example soon after. M. *Rolland* published a very able memoir on the subject, in the Medical and Surgical Journal of Toulouse, of August, 1837, in which he establishes the excellence of the principle for which I had contended—that of *debridemens multiples* of the prostate for the extraction of large calculi. He has reported several most interesting cases in which he succeeded in extracting large calculi by dividing the prostate in different directions."

*Amputation of Gangrened Limbs.*

M. *Vidal,* it will be found, adheres very nearly to the maxim of the older surgeons, not to perform the operation, until the line of demarcation between the dead and the living parts is pretty fairly established.

"A surgical law," says he, "as wise as it is old, lays down the precept that we should not have recourse to the removal of a gangrened part until after the appearance of the circle which separates it from the surrounding tissues. It has been thought by some that we need not adhere to it when the gangrene is of internal origin; but let the surgeon be on his guard; the infraction of the law, even under such circumstances, may induce the most disastrous results.

When a limb has been severely contused, how difficult it is to know what will be the limits of the mortification, and of the suppurative inflammation that may ensue. A contusion, which may be supposed to be confined to the foot, often extends up as high as the knee, for the ecchymosis does not in all cases manifest itself outwardly; and even the nervous commotion may compromise the life of the entire limb, although there may not be any distinct external symptoms.

We should, therefore, as a general rule, refrain from amputating a limb before the establishment of the inflammatory circle, except when the gangrene is very near to the trunk of the body, and the exhaustion of the system will not permit us to wait any longer. But, even under such circumstances as these, many a prudent surgeon will not consent to perform the operation, as it is then almost inevitably fatal."

The French reviewer thus comments on the preceding passage. "It will be perceived that M. *Vidal* is an exceedingly prudent practitioner, and, in a man who has not had the melancholy experience of his own failures to instruct him, this may certainly be regarded as a proof of superior judgment. We have no cause to fear that the study of his work will inspire young surgeons with temerity or presumption: it is in every respect *un livre sage*. Now this is the very quality most needful in works intended for the inexperienced, even although the *sagesse* may be sometimes carried a little too far."

*Hospital Gangrene.*

It is gratifying to find from the experience of military surgeons that this frightful disease is, like scurvy, becoming gradually less and less frequent; owing, we may hope, to the improvements which have taken place of late years in public *hygiene*.

While our author confirms the general accuracy of this statement, he adds, "I read, however, in the recent work of M. *Baudens* on Gunshot Wounds, that in the military hospitals at Algeria the disease has sometimes prevailed so extensively, and so fatally, that every patient, who had undergone an important operation, died." Whether this lamentable circumstance is attributable to the unhealthiness of the climate, or to some local disadvantages of the hospitals, does not sufficiently appear. *Delpech* defined hospital gangrene as "a peculiar disorganisation of the soft parts, so that they disappear without leaving any trace of their primitive tissue, and become a putrid homogeneous gluten." M. *Vidal* accepts this definition, but not without criticising it: "This definition," says he, "which has not only all the defects of surgical definitions, but also those which characterise the style of the author, may serve, however, to give an idea of the singular disease of which we are treating."

The blood-vessels, more especially the arteries, resist more than any other tissues the destructive corrosion of the disease. A case is mentioned by our author where a wound in the groin became affected with hospital gangrene; all the soft parts around, to a very considerable depth, were fairly dissected out, but the crural artery was spared. When the gangrene was once stopped, the rapidity, with which the enormous wound was filled up, was truly wonderful. M. *Vidal* very strongly recommends the application of nitric acid in cases of hospital gangrene, the wounds having been previously washed with a decoction of Provence roses in wine.—*Archives Generales de Medecine.*

---

### M. Sedillot on Surgical Instruction.

M. *Sedillot*,—after having distinguished himself as an energetic and enterprising military surgeon, first in Poland, during the last revolution in that unfortunate country, and subsequently in Algeria,—has been recently appointed the professor of surgery in the hospital at Strasbourg. The following passages are taken from the introductory lecture to his first course.

*Unity of Science.*

"The various divisions of science exhibit so many points of mutual contact, or rather they are so intimately and necessarily united together, that it is scarcely possible to study any one apart from the rest; all affording reciprocal explanation and enlightenment.

Mechanical and chemical philosophy serves to initiate the mind in the general laws of nature, and to disclose the operation of the chief external agencies which modify the functions of the living system.

General and descriptive anatomy reveals to the surgeon the unity of the entire organisation; the anatomy of the different regions of the body guides his hand; and pathological anatomy directs his judgment amidst the numerous morbid changes of the various tissues and organs. External pathology reveals the wounds and other lesions of outward parts; internal pathology those of the hidden parts; while the study of materia medica and of operative surgery supplies him with the means of relieving or curing them."

*Advantages of early Clinical Study.*

M. *Sedillot* is a zealous advocate for the student attending on hospital practice from the very commencement of his *curriculum.*

"I do not dispute," says he, "the advantage of knowing theoretically the facts which we have to observe; for the mind in this way seizes upon all the experience of former times, and there is certainly some truth in the paradox that 'we see well only those things which we know.' I will even admit, and that willingly, that the rapidity of a student's progress is generally commensurate with the instruction which he has previousy acquired.

But this is not the question; to put it forth in these terms is really to solve it. What we wish to know is whether the student, who does not begin his attendance on clinical practice till the third or fourth year of his studies, is likely to be as adroit at the diagnosis and treatment of diseases as he who has regularly attended to it from his first matriculation. The answer cannot be very doubtful; for the art of observing *ne s'improvise pas;* it needs long and assiduous practice; and if it does require a previous knowledge of general science, your literary and scientific diplomas sufficiently vouch for your competency to observe the leading phenomena of disease as revealed at the bedside of the sick. The majority of our students prefer, however, to follow the programme laid down by the university, and usually pass their first four examinations before commencing their clinical studies. The result of this practice is, that generally the fifth examination is *tres faible.*

During three years of service which I spent as an aggregate of the faculty of Paris, I have repeatedly observed that the students, who may have gone through their early examinations with éclat, scarcely knew how to interrogate a patient, and frequently mistook the most obvious symptoms and indications of a well-marked case. The diploma of doctor is, however, awarded to you upon the *ensemble* of your knowledge, and you are left to acquire subsquently from actual practice the experience in which you may be deficient; but, in your efforts to obtain this, you are necessarily exposed to the most cruel errors, and often run the risk of compromising your future peace and your success for life.

I readily admit that, in the short space of four years—the period that is judged sufficient for your medical education—the general study of the various branches of science, which compose the *curriculum,* must take the precedence of that of special facts; for the latter will be the business of your whole after-life. I do not advise you to neglect any of the courses prescribed; but it seems to me that clinical instruction might almost always be blended with their study at the same time. Few students begin their labours early in the morning; and perhaps all of you will admit that, in some way or another, you lose one, if not several hours in the course of every day, which might be well devoted to observation in a hospital. However this may be, you may be assured that this is the only sure manner of acquiring that ready tact which theory can never communicate.

That surgical *coup-d'œil,* which enabled such men as *Dessault, Boyer,* and *Dupuytren,* to discriminate from one end of a ward to another the cases of their various patients, belongs, no doubt, in a great measure, to individual aptitude; but still it mainly depends upon the constant study of actual disease. It is only by the union of great personal experience in the observation and treatment of disease, and of due acquaintance with the recorded experience of others, that the student can ever hope to follow in the footsteps of these great men. Never forget that, whatever may be your destiny, you will ever retain the remembrance of those facts and precepts which have been presented, so to speak, to your eyes, and in which you have taken a personal part, while it will require a constant effort to bear in mind such as have been addressed only to the ear. The maxim of *Horace* applies, in an especial manner, to medical studies:—

*Segnius irritant animos demissa* per aures,

*Quam quæ sunt oculis subjecta fidelibus:*

It is almost unnecessary to dwell upon this subject, as its truth is abundantly testified by the circumstance that your very co-students, in whom you have most confidence, are just those who become *internes* of the hospitals; and this situation, you are aware, can only be obtained after much diligence and long clinical attendance. An error, into which many of you are apt to fall, is from an over-zeal to observe and take notes of too many cases at the same time. This is not the right way to acquire a practical knowledge of diseases. While taking a general survey of all, let your attention be particularly directed to three or four cases at a time, reading at your leisure upon the diseases in question from such works as the treatises of *Sabatier, Boyer, Begin, Roche,* and *Sanson,* or of M. *Vidal.*"

*Influence of Constitution on Surgical Diseases.*

" Persons of the melancholic temperament—which is owing to a predominance of the venous system—are more subject than others to various sanguineous effusions; and one of my colleagues, M. *Judas,* has published in the Gazette Medicale an interesting memoir on sanguineous tumours of the labia, which he has found to be of frequent occurrence in women of this temperament. Swellings of the glands are rather rare in those of a plethoric or of a nervous constitution, but are very common in lymphatic individuals. It has been observed that persons, who are wholly uneducated and whose encephalon is but ill developed, are more than usually liable to be affected with typhus and to sink from the effects of it. Again, the more consistent and plastic that the blood is, the less tendency there is to re-actions of any sort. The Russian soldiers,—in whom hebetude of mind, a strong and gross diet, a cold climate, a native want of cleanliness, render the blood so thick that it adheres tenaciously to the fingers of the operator—are but little subject to fever after the most severe wounds. In the greater number of those, on whom I operated during the Polish campaign of 1831, the pulse was quite normal on the fifth or sixth day after the operation, and their appetite was as good as ever. On the contrary, in men who are much addicted to study, or who are of a highly irritable temperament, the blood is usually thin and not sufficiently viscid."

*Every Surgeon has his own Methods.*

" You will find it very useful to follow the clinical courses of several surgeons; for every one has his own favourite views and favourite methods of treatment. By taking notes regularly of the practice of each, you will gradually become capable yourselves of judging of their respective merits, and you will acquire an exactitude and a precision of knowledge that will be most valuable to you through life. It is often very difficult to ascertain the exact relations between the cures obtained and the methods of treatment employed; it is only by an accumulation of results, carefully and cautiously collected, that we can judge of the superiority of one method over another."

*Influence of External Circumstances on Wounds.*

" In the last expedition to Constantine, (in Algeria), I cured a great number of the wounded, in whom immense purulent collections had formed in the lower limbs, by prescribing freely brandy, coffee, fresh meat, &c. and by the use of warm dry applications. Be it remembered that the poor fellows had been lying in the mud for several nights, under a bitter cold atmosphere, and that their rations also had been curtailed in consequence of the deficiency of provisions. (A similar remark was made by *Larrey* and other surgeons of the French army during the disastrous campaign of 1814 in Germany.) Many a case of purulent infection may be prevented by the timely administration of an emetic, and the use of a nutritious and even a moderately stimulating diet. I wish particularly to guard you against the practice of specialising or localising diseases too much, and of supposing that, if you treat a wound locally well, you have fulfilled all the indications of enlightened surgery. Far from it; the good practitioner will never forget to watch the state of the general health, and endeavour to maintain the equilibrium of all the functions of the system."

*Familiarity between Students and their Teachers.*

"I shall take this opportunity of stating that it will always give me great pleasure to hear the opinions of any of you upon a case where they differ from those which I have formed of it. In this respect I wish to imitate the example set to hospital surgeons by that great master of our art, the late Baron *Dupuytren*. Although his professional and scientific susceptibility was excessive, he was not above accepting advice from whatever quarter it came. I remember a striking instance of this feature of his character. A patient, who was received into the Hôtel Dieu, was found on examination to present the symptoms common to a dislocation of the shoulder and to fracture of the cervix of the humerus. After delivering a most able lecture on the diagnostic symptoms between these two injuries, he confessed that in the present case he was a good deal puzzled, and that he intended to wait a day or two before determining the point. On the morrow, he received a long letter from one of the pupils of his clinique, in which the writer not only proved by very satisfactory arguments that the case was in truth one of luxation, but pointed out the best means of reducing it.

*Dupuytren* read the letter before his class, summoned the patient before him, discussed each argument separately with his youthful opponent, whom he had called to his side, and with his assistance reduced the dislocation amidst the loud plaudits of all the pupils."

*Injunction of kindness towards Patients.*

"You are about," says M. *Sedillot* with equal beauty and justness, "to find yourselves in contact with fellow-creatures afflicted with two of the greatest ills of humanity, pain and distress. They may often seem to you to be susceptible, irritable, and even unjust; but do not forget that they are your brethren in equality, and that the harder their situation is, the more it should commend your sympathy, and kind respect. Learn to forego the mere claims of science, and do not regard the unfortunates before you as if they were only objects for professional observation. Avoid especially the slightest indiscretion of language; and let the expression of your features, the sound of your voice, and every, even the most trivial, acts of your behaviour, always testify your entire confidence in the resources of nature, and in the remedial means of art. The unguarded disclosure of danger, or the allusion to possible accidents, has on many occasions produced the most disastrous consequences, and changed a case of promising success to one of rapid and irremediable danger."

*Impolicy of explaining too much to Patients.*

M. *Sedillot* condemns the not unfrequent practice of explaining to patients, in the event of an operation being necessary, the relative dangers attending its performance, and those of leaving the disease to itself.

"The surgeon ought not ever to dread the responsibility of his opinions or of his acts, if he obeys the dictates of a conscientious conviction: by acting otherwise, he erroneously supposes that he escapes the consequences of his conduct, and moreover he often greatly compromises his chances of success. By revealing to a patient the chances of recovery and the dangers which necessarily attend an operation, you plunge him into a state of dreadful anxiety, and in truth you leave him only the mockery of a free choice. How is it possible for any uneducated person to judge of a matter, of which you cannot explain to him even the very simplest elements? In my opinion, the surgeon's duty is, after basing his judgments on science, to perform without hesitation what appears to him to promise the best hopes of success to his patient, who will have much more confidence in his medical attendant, and more cheerful expectation as to the result, than by adopting a contrary practice."—*Journal des Connoissances Medicales.*

---

### Condemnation of Myotomy in Spinal Deformities.

M. *Bouvier*, we are glad to observe, in a memoir read before the Academy of Medicine in July last, and published in the Annals of Surgery for December, has exposed the utter fallacy and folly of M. *Guerin's* myotomic practice in cases of lateral deviations of the spine.

Our readers are probably aware that the "grand myotomiste" of Paris attributes almost all deformities whether of the trunk or of the extremities to a permanent contraction of certain of the muscles of the affected part; and, assuming this position as established, he very fairly concludes that, if these muscles are divided across, the deformity will necessarily be lessened, if not entirely removed. M. *Guerin* seems to regard the human body very nearly in the same manner as a sailor does his ship. When the latter wishes to square any yard, which is much aslant, he lets go on the lee side the ropes which are called the *braces*, and gives a pull upon those on the weather side. Just so does M. *Guerin* in his treatment of deformities; he cuts across the muscles and tendons which, according to his view of the subject, are *taught* (to use Jack's phrase), and looks for nature to give a pull upon the antagonist ones, that had been slackened.

If we could give credit to his own reports, his success has been truly marvellous; and certainly the rapidity with which, he tells us, many of his cures are effected, must add greatly to the value of his "belle decouverte." So simple, he goes on to say, is the operation which he performs, that he has divided nearly 40 (!) muscles or tendons, (according to the sub-cutaneous plan), in one patient at a single sitting. Good news for all the wry-backed, stiff-necked, club-footed people of the world! it is their own fault if they remain one day without being made as straight and upright as He of the silver bow.

It is certainly a curious feature of the surgery of the last few years, this passion for dividing muscles and tendons, or for myotomy, as the operation has been called. We suppose that all the squinting people in Europe have been cured by this time; and as for club-feet, they are never heard of now. Poor *Byron!** why were you born before the present day? Many a vexatious moment might have been spared thee, had the star of thy nativity not arisen until the fourth decenniad of this century. But, alas! the fates are very wilful, and too often make use of any disease or deformity in the body to whip weak man for the follies or vices of his mind.

---

* It has been remarked as a curious circumstance that three of the most distinguished men of the present century were club-footed—*Byron, Scott,* and *Talleyrand.*

To revert to the subject from which we started, we are glad to find that some of the French surgeons themselves are inclined to oppose the absurd practice to which we have been alluding. M. *Bouvier*, after detailing several experiments on the dead body with the view of ascertaining the truth of M. *Guerin's* theory of spinal deformities, very pertinently remarks:

"These facts speak for themselves. Since the resistance of the spine remains the same after the division of its muscles, and since, on the contrary, this resistance becomes small, when the ligaments are divided, although the muscles are intact, it is evident that these latter cannot be taken into account; and we scarcely require any other arguments to prove the dissimilitude between curvature of the spine and genuine muscular deformities—in which the resistance ceases, at least in a great measure, after the muscles have been divided, whereas the section of the ligaments produces little effect, if the muscles remain intact. The fundamental character of spinal deviations is unquestionably attributable to a lesion of the osteo-ligamentous apparatus of the vertebræ, and not to a mere spasmodic contraction of the dorsal muscles.

But, independently of the proofs furnished by post-mortem examinations, it is an easy matter to satisfy ourselves that during life there is not any tension or resistance of the muscles on the concave side of a deformed spine. We have only to be on our guard not to let ourselves be imposed upon by the contraction of these muscles to make us believe that there is any permanent shortening of their tissue. When the person is standing up, the contracted muscles of the back counterbalance the weight of the trunk, and they may therefore exhibit at that time a degree of tension proportionate to their physiological shortening. Now this tension is most decided on the convex side of the curved spine, in consequence of the body leaning over to the opposite side. One would need to be strangely pre-occupied with a favourite idea to attribute this condition of the muscles on the convex side to their abnormal retraction. If we try to bend the trunk in the opposite direction, then the muscles of the concave side appear to be stretched, because they then make an effort to maintain the equilibrium of the body."

M. *Bouvier* closes his memoir with the following two conclusions:

1. The section of the muscles of the back is absolutely without an object, and cannot produce any useful result in deformities depending upon a lateral deviation of the spine.

2. The treatment of such deformities must be based on remedying the shortened condition of the concave side of the spinal column, its muscles always retaining sufficient length not to present any resistance to the *redressement*.

Here might terminate the examination of this question for any one who will think for himself and study the facts which we have mentioned without any prepossessions or after-thoughts.

But M. *Guerin* and his disciples, ill at ease in the circle of positive pathological facts, have appealed to the results of their experience and the success attending their practice—a species of argument which is certainly very convenient, since it dispenses with every other. But even on this ground we are satisfied that they are completely at fault, that their vaunted successes have nothing real in them, and that experience will agree with theory in condemning the division of the muscles of the back in the treatment of spinal deformities.—*Gazette Medicale.*

---

### M. SERRE ON THE INFLUENCE OF THE OPERATION FOR CATARACT IN ONE EYE ON THE SIGHT OF THE OTHER.

In 1834 I operated for cataract on the right eye of a patient, on whose left eye *Delpech* had operated three years previously. For two years and a half after this first operation, he retained the sight of the left eye; but, during the latter six months, it had become more and more weak, until it was entirely lost.

A few days after the second operation however I was not a little surprised to find that the patient had recovered the sight of both eyes. At first I hesitated to believe it; but, on cross-examining the patient, his statement was uniformly consistent.

Shortly after this case occurred to me, another cataract patient was admitted into the hospital, in nearly the same condition as the former one. He had been previously operated upon for a cataract in the left eye by an itinerant oculist, and had regained the sight of the eye for some time, but eventually he had again lost it. I couched the right eye; and the result was, that the vision in both eyes was restored. Unfortunately this double success was not of long duration; for the recently operated eye became severely inflamed, and at length its pupil was obliterated. The sight of the right eye nevertheless remained, although it had been lost for nearly twelve months before the second operation.

For seven years I did not meet with a case similar to the preceding ones, although during that time I must have operated on upwards of 200 cataract patients. In July of last year, on taking charge of the eye-patients in the hospital, I found a man on whose left eye M. *Lallemand* had operated about two months before, but who had not yet regained his sight, in consequence it seemed of that sort of amaurotic condition which sometimes follows the depression of the lens. There was nothing unusual in the appearance of the pupil, and no symptom of inflammation existed; and yet the patient was so blind that he could scarcely distinguish light from darkness. I determined therefore to operate on the other eye; and on the 14th of July I accordingly performed the retroversion of the right lens. From the fourth day after the operation, the patient could perceive objects, and I very naturally attributed this recovery of sight to the operation; but this was a mistake; for on examining the right eye the displaced lens was observed to have re-ascended. The truth was, that it was the other, the left, eye that the patient was able to see with.

As soon as the inflammatory symptoms caused by the operation had subsided, I again displaced the right lens, and lodged it fairly in the substance of the vitreous humour. In proportion as the sight of this eye became stronger, that of the other eye continued to improve; so that when the patient left the hospital on the 1st of September, he could see pretty distinctly with both eyes.

It may be asked how are we to explain this curious circumstance? Let us first observe that the three patients alluded to in the preceding remarks had, each of them, recovered for a short time the sight of the eye first operated upon, although they subsequently lost it again; and that when the other one was operated upon, it (the former one) still retained its transparency, as well as the regularity of its pupil, so that the rays of light reached the retina without obstruction. The sensibility of this nervous expansion seems to have been only temporarily paralysed, and to have been in such a condition as to require for its re-excitement merely that a lively impression should be made on the retina of its fellow.

Let it be remembered that such cases as we have now described are very different from those which we find recorded by some oculists, and in which we are told that patients with double cataracts have recovered the sight of both eyes after the removal of one of the lenses. We are unwilling to deny positively the truth of all such statements; although it must be confessed that they appear rather marvellous.

On the contrary, if cases, similar to those I have observed, are occasionally met with in practice, may we not ask if it might not be possible, in the treatment of certain amauroses, and more especially in the asthenic form of the disease, to avail ourselves of the light as a stimulant, where the loss of vision is on one side only? Again, far from abstaining to operate on a cataractous eye when the patient sees but feebly with the other one, may we not in certain circumstances try the operation with the express view of re-awakening the sensibility in the latter? And lastly—contrary indeed to the opinions of many oculists—may not the excitement produced by the operation or by the direct action of the light serve, in some cases of cataract complicated with incipient amaurosis, to contribute to the restoration of sight?

These are questions well meriting the attentive consideration of every scientific ophthalmologist.—*Gazette Medicale.*

---

### M. SERRE ON THE INFLUENCE OF INFLAMMATION IN ONE EYE ON THE RESTORATION OF SIGHT IN THE OTHER.

The following two cases are interesting, and contribute to throw some *light* on the preceding article. They are communicated to the Gazette Medicale by M. *Serre,* the Professor of Clinical Surgery at Montpelier.

*Case* 1.—While treating one of the cases mentioned in my former paper, I had under my care a patient in the left of whose eyes the pupil was obliterated, and in the other one there was an almost complete amaurosis. As all the means, which I had tried to relieve the blindness, had proved quite ineffectual, I determined to try to form an artificial pupil in the left eye. With this view I made a small incision about the centre of the cornea, and introducing the hook used by *Beer,* I laid hold of the iris near to its point of junction with the ciliary body, and detached it in such a manner that I could lodge it in the opening of the cornea, and thus permit a passage to the rays of light to reach the bottom of the eye.

This operation succeeded beyond my expectations; as far at least as regarded the establishment of an artificial pupil; for this continued to preserve its original dimensions; but, from the moment that I first detached the iris, I perceived that the deep parts of the eye were too much injured to permit me to anticipate a recovery of sight. The inflammation however, excited by the operation, had this good effect, that the sight of the other eye was very sensibly improved. Alas! however, this was only a deceitful promise; for, as the inflammatory symptoms in the one eye subsided, the sight of the amaurotic eye gradually decreased, until the patient was as blind as ever.

*Case* 2.—A patient, under my care, had a cataract in the right eye and a strongly marked amblyopia in the left one. I operated for the cataract, and it was observed both by myself and by several of the attending students that the sight of the left eye was very considerably improved by the inflammation which was set up in the other. The operated eye became subsequently affected with purulent ophthalmia, in consequence of the patient having imprudently exposed himself to cold, and was at length irrecoverably lost; but fortunately this accident, so far from injuring the improvement in the amaurotic eye, seemed actually to have confirmed it more.

Reasoning upon the facts now mentioned, I tried the effect, in one case of amaurosis, of introducing a cataract-needle into the posterior chamber of the eye, so as slightly to titillate the portion of the retina at the inferior part of the eye. A very trifling result, and that was of only temporary duration, followed the attempt; probably the degree of inflammation induced was not sufficient for the purpose.

When I consider the good effects which the application of caustic to the cornea, or even the mere repeated friction of the eyelids, produces in some cases of amaurosis, I am much inclined to hope that something may yet be discovered in this direction for the relief of this most unfortunate malady; and that it may be from our not yet understanding how to accommodate our remedies to each case, that hitherto so little success has attended our labours in this department of ophthalmic medicine. Instead of acting merely on the teguments of the eye and on the cornea, we should have our attention directed to the frontal nerve, to the ciliary ganglia, the iris, and even to the retina itself, before abandoning a case as utterly hopeless. As a matter of course, it is only in cases where the humours of the eye retain their transparency that the suggestion now thrown out is meant to be at all applicable.—*Gazette Medicale.*

---

### Review of the Ophthalmological Literature of 1840 and 1841.

### *Fistula Lachrymalis.*

M. *Rognetta* objects in strong terms to the very mechanical mode of treating this disease, that is too generally followed, and suggests another which, in his hands at least, has superseded, he says, the use of the knife, as well as of probes, canulæ, &c. afterwards. Certain it is that a number of cases do seem to be rather aggravated than amended by the usual practice. Let us consider for a moment what this is. First of all leeches are applied; then cataplasms, then fumigations by the nostrils, injections by the lachrymal puncta, and the application of astringent lotions or ointments are successively had recourse to. Finding, perhaps, that no good has been obtained, the surgeon now resorts to the use of the knife, and of the canula; this latter instrument generally requires to be withdrawn after a short time, and the wound contracts or may close up entirely. A second operation is then deemed necessary; and perhaps this one does not prove more successful than the first.

"I have long opposed," says M. *Rognetta,* "this mode of practice as being alike unscientific and most unsatisfactory. Often it exasperates the disease; and in other cases it only palliates without curing the evil. As long as surgeons view fistula lachrymalis as nothing else but a mere mechanical obstruction, we cannot expect any improvement in the mode of treating it. The canal is stopped up, they tell us, and we must try to open it and keep it so. Hence has arisen the use of sounds, canulæ, permanent setons, and all such mechanical contrivances.

In vain the system re-acts against this mode of treatment in a variety of ways; and often the disease is reproduced again and again; but the surgeon closes his eyes, and regards the case merely as one of more than usual obstinacy. Unless the dynamic state of the disease is understood and attended to, we cannot expect any improvement in our practice. Whoever has examined in the dead body the condition of the lachrymal organs in a state of disease, must have observed that the canal for the passage of the tears into the nostril is obstructed by the swelling of its mucous membrane, especially of its follicles, and by a sort of thickening and infiltration of the sub-mucous tissue. Is it not obvious that whatever increases irritation must necessarily aggravate the very existing mischief? For some years past I have acted on the principle of endeavouring to remove the inflamed condition of the mucous membrane, and to correct the secretion of its follicles, and have met with a most pleasing success in eight or nine cases of the disease."

M. *Rigaud* concludes some observations on the same complaint by remarking that, "in young subjects a great number of lachrymal tumors become spontaneously cured." The improvement of the general health and the development of the organs at different periods, seem to him to have a much greater influence than the use of fumigations, lotions, &c.

Dr. *Ridder* relates a case in which the use of chloride of lime injections, frictions with calomel ointment, fumigations with strong chamomile tea, and the administration of mercurials internally, effected a cure of a case which had long resisted previous treatment.

*Wound of the Orbit; Rupture of the Optic Nerve.*

Mr. *Phillips* has recorded, in the London Medical Gazette for January 1841, a curious case of this very rare injury. A man, standing at the head of a horse which had fallen in the street, was suddenly struck in the face upon the animal raising itself up unexpectedly: the blow was so violent that he was thrown down by it. He was of opinion himself that it was not the head of the horse, but some part of the harness that struck him. There was a bleeding wound between the *left* eye and the nose, extending for about three-quarters of an inch from the internal canthus to about an inch below the eyebrow. The lachrymal ducts and the tendon of the orbicular muscle were divided across; but the eyeball had not suffered. The sight of the opposite or the *right* eye was lost from the moment of the accident; and yet no alteration could be perceived in any part of it, except extreme dilatation of the pupil, which did not contract even upon the approach of a lighted candle. The patient complained of slight headache, but nothing indicated the existence of any lesion within the cranium. Delirium, however, and stupor supervened on the following day; and, as these symptoms were attributed to the invasion of meningitis, the patient was accordingly bled, purged, and treated with repeated doses of calomel and antimony. In the evening convulsions came on; while the left arm and leg were stiff and contracted, the right extremities were in constant motion; the pupil of the right eye was now found to be contracted. As the patient could no longer swallow pills, calomel was applied on the tongue; a blister also was applied to the nucha. The left side and extremities became subsequently paralytic, while the right were tranquil. He died convulsed on the fifth day after the accident.

*Dissection.*—There was a marked vascularity also and a copious effusion of lymph between the arachnoid membrane and the pia mater. A quantity of serum and pus was found in the lateral ventricles. Upon lifting up the anterior lobes of the cerebrum, they were observed to adhere by their lower surface to the dura mater in consequence of effused coagulable lymph. The right optic nerve was found to be fairly torn across; the two ruptured ends adhering together only by a thin membrane, close to the optic foramen. The base of the brain, from the medulla oblongata to the commissure of the optic nerves, was invested with a thick covering of plastic lymph, which partly concealed the roots of the nerves. At the posterior part of the right anterior lobe, and near to the seat of the lacerated nerve, there was a small spot where the cerebral substance was in an ecchymosed and softened state. This injury of the encephalon, as well as the laceration of the nerve, had been caused by a spicula or fragment of bone, detached from the circumference of the optic foramen. Upon examining the orbitar wound attentively, there was found a small aperture, by which a probe could be made to pass through the breach in the ethmoid bone into the cranium. This shewed that the instrument, which the horse's head had driven in the direction of the opposite orbit, had been pointed, and that it must have struck with force on the *os planum,* passing from below upwards to the cerebral lamina of the ethmoid bone of the opposite side.

Dr. *Rognetta* appends the following observations to the history of the preceding case.

"Although there are several analogous cases recorded in surgical works, the present one is in some respects almost unique. The most remarkable circumstance connected with it is the direct lesion of the optic nerve of the side opposite to that of the wounded orbit. We know that the optic nerve may be wounded directly in the orbit by a pointed instrument entering by its external canthus; for, as it describes a curve with its convexity outwards, it is readily accessible from this part. But, before the case related by Mr. *Phillips* was made known, we had never heard of an injury of the intra-cranial portion of one optic nerve by an instrument which had entered by the internal canthus on the other side.

It is worthy of notice that in this, and in other somewhat similar cases where the optic nerve alone has been injured, the ball of the eye usually does not exhibit any outward marks of the lesion; the only symptom present being amaurotic blindness. In a dissection made by *Cheselden* and in another by *Morgagni,* the optic nerve had been for a length of time disorganised from spontaneous disease; and yet the eye in both instances retained its normal features in every respect. Do not such facts shew that the optic is purely a sensory nerve, and has nothing to do with the nutrition of the eyeball?

*Dupuytren* used to mention the case of a fencing-master, who met with his death in the following manner. His adversary's foil, though guarded with a button, pierced through the wire-fence of his masque, and struck him at the base of the right upper eye-lid, making a small wound there. He fell down, and was carried to the Hôtel Dieu. On the morrow, alarming encephalic symptoms, delirium, convulsions, coma and fever, supervened, and he died two days afterwards. On dissection, the orbitar plate of the frontal bone was found to have been pierced by the point of the foil, which had penetrated so deep as to wound the anterior lobe of the brain. Another case, very similar to this one, occurred to one of the pupils of the Polytechnic School; he remained hemiplegic.

The same sort of accident has been known to be caused by a blow with the point of a cane, of an umbrella, of a fork, of an awl, &c. &c. In a few rare instances, the optic nerve has been lacerated by a violent luxation of the eye-ball itself.

### *Trichiasis.*

In the Annales d'Oculistique there is a memoir by M. *Bourjot* on the different operations which have been recommended for the cure of this deformity. He gives a decided preference to that proposed and practised by M. *Jaeger,* and which has been adopted with success by M. *Cunier.*

It consists in making a horizontal incision of the skin of the eyelid near to its free border, carefully exposing the bulbs of the cilia and then excising them. M. *Bourjot* directs the attention of his readers to the promptitude with which supernumerary cilia are developed on the internal surface of the eyelid, and to the difficulty often experienced in exposing those that are diverted from their natural position.

A case, related in the Netherlands Lancet, shews with what care the eye should be examined, when it is affected with a chronic disease. A poor fellow had been suffering for several successive months from a most painful ophthalmia, attended with great photophia and an excessive flow of tears, when he applied for relief to Dr. *Snabilie,* the surgeon major of Breda. A minute lash had become inverted and kept up a constant irritation on the eye; no sooner was this extracted than all the symptoms vanished.

### *Ptosis.*

Dr. *Alessi* has recorded in an Italian Journal, the Filiatre Sebezio, a curious case of hereditary falling down of the upper eyelid in several members of a family in Sicily. The man, who first applied to him, was affected with an incomplete ptosis of the left upper eyelid; it was more considerable at the outer than at the inner canthus. When he wished to look at an object with this eye, he was obliged to turn his head round over his right shoulder. On being questioned how this malady had occurred, he told Dr. A. that it was hereditary in his family, for that both his father and his son were affected in a similar manner.

By a bizarre singularity, the males alone were affected; and what makes the occurrence still more strange, is that the deformity changed sides at each generation. Thus in his father's case it was the right eye that was affected; in himself, it was the left one; in his son it was again the right; and in his grandson it was the left. Dr. *Alessi* satisfied himself of the truth of the statement by personally examining his son and grandson. He (the Dr.) was of opinion that the falling down of the eyelid was owing, not to any paralysis or atony of the levator muscle, but to an unusual flatness or depression of the supra-ciliary arcade of the frontal bone, so that the integuments, although not abnormally lengthened, hung down in front of the eyeball. Dr. A. proposed an operation, but none of the patients would submit to it.

*Mechanical Lesions of the Eye.*

Dr. *O'Beirne* has published in the Dublin Medical Press a curious case in which a small nail was accidentally driven into the eye-ball, and lodged there for many days.

The patient, a woman, said that, while shaking a carpet, she felt something sharp strike with force against her right eye. She became sick immediately, and shortly afterwards she found on her apron a gelatinous substance, which is supposed to have been the lens. When admitted into the hospital, there was so much tumefaction and ecchymosis of the eye that the cornea could scarcely be perceived, except at one point, where there was seen to be a depression, from which a bloody fluid oozed out. There was no appearance of any foreign substance in the eye; and indeed the woman herself said that the nail had been found on the carpet. In spite of the most active antiphlogistic treatment, the inflammation and suffering increased for nearly a fortnight: and then an eschar formed about the centre of the cornea. Upon making a puncture there, a considerable quantity of purulent matter flowed out with decided relief to the symptoms. Dr. O'B. while making the puncture, thought that he felt the point of his lancet strike upon a hard substance, and therefore suspected that something was lodged in the eyeball. On the following day, his suspicions were confirmed; and he then extracted, not without some difficulty, a flat-headed nail of about three quarters of an inch in length. The inflammation quickly subsided; but, as a matter of course, the sight of this eye was irrecoverably lost.

MM. *Cunier* and *Stievenart* have related cases in the first vol. of the Annales d'Oculistique, where fragments of fulminating capsules had been driven into the eye. In one case an entire capsule was extracted between two and three months after the occurrence of the accident.

*Wounds of the Supra-ciliary Region.*

M. *Constatt* has, in the first volume of the Annales d'Oculistique, established by numerous historical and necroscopic researches that the blindness, which sometimes follows wounds of the supra-ciliary region, is, in almost every case, owing to some other cause than to an injury of the frontal nerve, as is usually imagined. M. *Walther*, in a recent number of the Journal der Chirurgie und Augenheilkunde, alludes to several cases in which no blindness occurred, although this nerve had been positively divided either accidentally, or designedly, for the relief of neuralgia.

When loss of sight follows wounds about the forehead, he is inclined to attribute it to some simultaneous derangement of the organs contained within the orbit or the cranium, and not to any direct injury of the frontal nerve.

M. *Walther* endeavours to shew that there is no direct communication between the frontal nerve and either the optic nerve or the retina; that even with the ciliary system of nerves its communication is only indirect through the medium of the nasal nerve; and that impressions on it, (the frontal), are transmitted to the eye through the medium of the encephalon. According to this view, there is therefore no direct, but only a reflex, continuity of action.

The nutrition of the eye is disturbed by any lesion of the ganglionary nervous filaments, which are distributed on this organ. Thus diseases of the neck, or operations performed in this part, will sometimes produce ophthalmia, or even an atrophy of the eye. If, then, says M. *Walther*, lesions of the great sympathetic nerve have so marked an effect on vision, why should not an injury of a branch of the trigeminus, which is well known to be so intimately connected with the eyeball, produce the same results?

The French Medical Gazette adds to its analysis of M. *Walther*'s paper a case where blindness followed a slight wound of the forehead, although there was no obvious commotion either of the eyeball or of the encephalon. The blindness in this case was owing not to amaurosis but to the presence of a cataract: in consequence probably of the nutrition of the eye being disturbed.

*Purulent Ophthalmia of Armies.*

For the last few years the Belgian army has suffered from an unusually severe epidemic of purulent ophthalmia, which has produced very disastrous effects on numbers of the soldiers who have been affected with it. A vast number of memoirs and books have already been published not only in Belgium itself, but in other countries, as in France, Germany, and Russia, medical men from which have been specially sent by their respective governments to report upon the disease. As might be expected, there has been no little difference of opinion as to the cause and nature of the ophthalmia in question; some writers contending that there is something specific in its origin and character, and that, differing in these respects from all the ordinary forms of the malady, it is essentially allied to those frightful epidemics which in all ages have afflicted the land of Egypt; while others asseverate with as much zeal that it is only a more than usually severe form of catarrhal or purulent ophthalmia.

The former view the disease as essentially contagious, and account for its wide diffusion on this principle: the latter hesitate to admit this doctrine, at least to so great an extent as has been alleged.

Dr. *Werneck* of Saltzburg, a high authority on ophthalmology, is a zealous advocate of the first set of opinions; and, after adducing many arguments to prove the essentially specific nature of the ophthalmia, from which the Belgian army in the present day, and the French and British armies about the beginning of this century, have suffered, he insists very particularly on the necessity of attending to the state of the eyelids in all patients affected with the disease;—for it is they, says he, that are "le veritable terrain où l'ophthalmie Egyptienne germe et developpe successivement tous ses effets."

M. *Decondé* has written some valuable papers on the different kinds of purulent ophthalmia. His researches have been directed in an especial manner to ascertain the effects of various remedial applications when blended with any contagious purulent discharge, whether from the eyes or from the genital organs.

In reference to the matter of gonorrhœa, the following are the conclusions to which he has come: 1. The discharge ought to be stopped as quickly as possible, as sooner or later it may produce a most severe purulent ophthalmia, if the matter be accidentally applied to the eye: 2. A strong solution of the nitrate of silver has the effect of altering the naure of the discharge, and destroying its contagious virulence: 3. The gonorrhœal discharge being the result of a morbid state of the mucous membrane more deep-seated and more enduring than that induced by injections into the urethra, it is always well to continue the use of these remedies for several days after the cessation of the discharge; otherwise this will most probably re-appear and resume all its characters.

With respect to purulent ophthalmia, M. *Decondé*, after detailing at great length a number of experiments with chloride of lime and other agents, infers that healthy troops, if brought into contact with an infected corps, may be preserved from the disease by the soldiers washing their eyes several times in the course of the day with a solution of chloride of lime.* This simple suggestion certainly deserves to be fairly tried; as no possible harm can result from the use of so simple a means.

On the whole, M. D. is decidedly friendly to the *ectrotic* method of treating all forms of purulent ophthalmia by the application of the nitrate of silver to the conjunctiva, more especially to that of the eyelids. M. *Goozée*, another writer in the Annales d' Oculistique, "gives a decided preference to this practice, and exposes the utter insufficiency of antiphlogistic measures, however vigorous, to arrest the progress of the disease."

M. *Ricord*, also, approves of the use of the nitrate, along with the application of poppy fomentations, and of extract of belladonna mixed with blue ointment. Local bleeding also may be necessary at the same time.

Dr. *Hancke*, of the Prussian army, recommends very strongly the application of iodine and of some of its preparations to the diseased surfaces: he is opposed to the use of copious depletion, and of drastic medicines.

*Purulent Ophthalmia of New-born Infants.*

M. *Reveille-Parise* tells us, as the result of his experience in this disease, that leeches, blisters, and emollient applications to the eyes are rather hurtful than advantageous; and that by far the best mode of treatment consists in the repeated instillation of a solution of the nitrate of silver—from two to four grains of the salt to an ounce of fluid—along with the exhibition of mild purgative medicines.

M. *Cunier* is equally favourable to the employment of the nitrate, but he prefers a much stronger solution, or, what he considers still better, an ointment made by blending the salt with lard. He advises that the action of the application should be confined chiefly to the conjunctiva of the palpebræ. M. *Cunier* states, as the result of his extensive experience, that in by far the greater number of cases of purulent ophthalmia in new-born infants, the disease is attributable to the direct application of leucorrhœal matter to the eyes during the act of delivery.

Dr. *Durre* of Halle employs an ointment—composed of nitrate of silver two grains, acetate of lead three grains, and lard three drachms—also blisters behind the ears, and a weak solution of the corrosive sublimate—one quarter of a grain to an ounce of water. He recommends also that frequent doses of calomel and rhubarb should be given at the same time. When the purulent discharge diminishes and gives way to one that is more watery, an eye-wash with the laudanum liquidum of Sydenham is one of the best applications.

Dr. *Rupp* tells us that he trusts chiefly to the use of a solution of the corrosive sublimate, one grain to the four ounces of water, as a topical remedy in the treatment of purulent ophthalmia in infants.

M. *Schwarz* recommends the application of strong tartar-emetic ointment to the nape of the neck, to produce a full crop of pustules, with the view of drawing the morbid action from the eyes.

*Scrofulous Ophthalmia.*

M. *Negrier* has, in a very able memoir published in the Archives Generales

---

* The solution of the chloride of lime has, according to the experience of M. *Decondé*, the same counteracting and neutralising effects on the matter of gonorrhœa. How far we can credit his assertion that "the gonorrhœa and the ophthalmic mucus may be deposited with impunity between the eyelids or in the urethra, provided the operation be preceded or followed immediately by the instillation of the chloride," we must leave our readers to determine for themselves.

for last May, directed the attention of the medical profession to the remedial powers of various preparations of walnut-leaves in scrofulous disease. The infusion and extract are the best forms for internal administration; and a very useful collyrium is prepared from the former, to which the tincture or the wine of opium should be added. Iodine or some of its preparations may be advantageously given at the same time.

The muriate of barytes, as an internal remedy, is highly spoken of by Dr. *Payan* of Aix: he considers it as one of the most valuable anti-scrofulous remedies that we possess; more especially in such cases where much irritability or erethism exists in the eyes or elsewhere.

Dr. *Otto* has published several papers in *Caspar's* Wochenscrift to prove the efficacy of the extract of conium, administered in tolerably large and gradually increased doses.

Dr. *Erdman*, in Graefe and Walther's Journal, dwells at some length on the excellent effects of quinine in some of the most obstinate cases of scrofulous ophthalmia. His practice agrees in this respect with that of many of the British oculists.

*Syphilitic Ophthalmia.*

M. *Sichel* says that his subsequent experience, since the publication of his work on diseases of the eyes in 1837, has quite confirmed the accuracy of the description which he then gave of the venereal form of iritis, and of the diagnostic peculiarities by which it may be recognized. He insists particularly, 1, on the discolouration of the iris, sometimes in its whole extent but always at its inner circle or circumference, to a coppery red or to a violet hue, and, 2, on the greater degree of tumefaction of this inner circle, than in the other kinds of iritis.* These two characters, (which, he says, are *constant*,) are owing to a particular kind of vascularity in the smaller circumference of the iris. They are usually first perceived at its upper and inner corner; and even in the advanced stages of the disease, they are most conspicuous there. The pupil at this part is generally angular or jagged from being irregularly drawn upwards and inwards. This appearance is owing, at least in some cases, to an adhesion from deposited lymph between the pupillary margin and the crystalline lens. Although we cannot give any satisfactory reason for this part of the iris being more inflamed and more liable to be the seat of effused lymph than other parts of its circumference, the circumstance that such is really the case seems to be well established by the observations of M. *Sichel*. The irregularity of the pupil now mentioned, although not of constant occurrence, is observed in the majority of cases of syphilitic iritis, and is therefore a valuable diagnostic symptom, although certainly not so much to be relied on as the two former—viz. the peculiar discolouration and tumefaction of the iris. Occasionally the circumference of the pupil exhibits minute elevations or granules of a yellowish hue, on which may usually be perceived dark looking blood-vessels: these have been compared to condylomata in other parts of the body.

This form of iritis may always be regarded as a symptom of constitutional secondary or tertiary syphilis. We may be assured that the patient has had primary sores, although perhaps none of the usual secondary symptoms have subsequently appeared.

Surgeons may differ among themselves as to the uniformity of the morbid appearances of the iris, which are so much relied on by M. *Sichel* as characteristic of the syphilitic form of iritis; but that they are of sufficiently frequent occurrence to merit scrupulous attention will not be disputed by any. Let it be re-

---

* The iris seems, says M. *Sichel*, as if it was thickened by a tomentose or flocky layer of matter deposited upon its surface, especially near its pupillary margin.

membered that we are not to look for them at the very commencement of the disease; for at first the iris is observed to be only somewhat discoloured, but without exhibiting a coppery hue; nor does it yet shew any tomentose appearance; the pupil also retains its circular shape, and is only somewhat contracted and less moveable than in health. But, if an anti-syphilitic treatment be not adopted, the characteristic phenomena described above will inevitably be developed.

M. *Velpeau* has, we should state, questioned the accuracy of all M. *Sichel's* statements, and very positively asserts that there are no appearances which are at all characteristic of the syphilitic form of iritis during the acute stage of the symptoms; and that it is only when it has passed into the chronic stage that they are of any value. A case of acute iritis being given, there is not a single symptom, according to him, which can be said to distinguish one form or kind of the disease from any other.

*Occasional Forms of Ophthalmia.*

Inflammation of the eye has been occasionally observed to assume quite an intermittent character, and to require the use of bark or other anti-periodic remedies for its cure.

There is a very dangerous form of the disease which seems to be connected with a vitiated state of the fluids; it was noticed in several instances during the prevalence of the epidemic cholera, and was usually accompanied with ulceration of the cornea. In some cases, the humors of the eye escaped, and the organ was immediately destroyed.

Another very unfavourable kind of ophthalmia has been occasionally seen to occur in puerperal women, more especially after inflammatory attacks of the abdomen soon after delivery.

M. *Constatt* has described in the Annales d'Oculistique a troublesome form of the disease, which he has observed in several women during lactation. "It is announced by an irritation and subsequently a congestion of the conjunctiva, assuming the characters of a catarrhal, and sometimes also of a vesicular, ophthalmia. Occasionally it makes its appearance after some rheumatic affection of the joints or eruption on the face. From the conjunctiva the inflammation usually spreads to the cornea, and is accompanied with oppressive and darting pains in the eye and supra-orbital region; there is seldom however much intolerance of light. In all the cases observed by M. *Constatt*, an abscess formed in the cornea, usually about the centre of it. He is inclined to attribute this peculiar ophthalmia to an impoverishment of the blood from over-suckling, and recommends therefore in the treatment of it an abstinence from all depletions. The use of blisters behind the ears, and of mild cordial tonics, combined perhaps with colchicum or guaiac, internally, is recommended by him. The application of a solution of the nitrate of silver, alone or with laudanum, is the best local remedy. As a matter of course, the woman should cease suckling, and live on a generous, but unirritating, regimen.

It may be mentioned here that M. *Magendie* states that he has often observed an ulcerative ophthalmia in dogs, which were kept half-fed for a length of time, or into whose veins water or serum had been injected. The same circumstance has been noticed in patients exhausted from profuse losses of blood, long-continued purulent discharges, &c.

*Opacities of the Cornea.*

Several cases of the successful treatment of the *pannus cellulosus* with the cod-liver oil are recorded by MM. *Cunier* and *Delcourt.*

The *pannus vascularis* is often induced and kept up by a granulated state of the mucous surface of the eyelids: when this is rectified, the former disease is generally observed to diminish of itself. Signor *Riberi* of Turin has recently published a monograph, entitled, *Della ceratitide prodotta dalla degenerazione granellosa della Congiuntiva Palpebrale, Con osservazioni.*

MM. *Cunier* and *Hairion* report favourably of the use of the hydriodate of potash in some opacities of the cornea. The occasional application also of the solid nitrate of silver has succeeded in a good many cases.

*Staphyloma.*

Several cases of pellucid staphyloma, in which the cornea is abnormally protuberant but without losing its transparency, have been recently recorded in various medical journals. The evacuation of the aqueous humour, the occasional gentle application of the nitrate of silver to the sclerotic coat or the inside of the eyelids, compression of the eyeball, the use of sternutatories, blisters, &c. have been recommended in the treatment of the disease.

There is a form of staphyloma in which the cornea becomes so soft and yielding that it retains the mark of any impression made upon it. There is always a greater or less opacity in such a state of things; the disease seems to be owing to a softening of the texture of the cornea. It is described by *Rosas,* in his Handbuch der Augenheilkunde, under the name of *Atony of the cornea.*

M. *Delvigne* has described a case in which there was a small transparent tumor or vesicle situated on the cornea a little to the outside of its centre.

The opaque or ordinary form of staphyloma is unfortunately a not unfrequent result of epidemic and purulent ophthalmia.

M. *Ousenoort,* the distinguished oculist of Holland, has described in his journal the mode he adopts in removing the diseased cornea. M. *Guepin* prefers the actual cautery to the knife in certain cases, and reports several instances in which he employed it.

*Formation of an Artificial Pupil.*

M. *Sichel* is inclined to attribute the frequent failure of this operation rather to the bad selection of the cases for its performance than to any defect or insufficiency in itself. The great difficulty lies in knowing how to adapt it to each individual case, and also when not to undertake it at all.

According to this experienced surgeon, one of two methods will suffice for performing this operation in every case; provided there is not a complete opacity of the cornea, disorganization of the iris, or other serious lesion of the eye, which must render all operations utterly fruitless. These two methods are—1, the detachment of the iris (*iridodialysie)* and 2, excision of part of it (*iridectomie*).

The former may generally be practised whenever we have to establish a pupil at the upper and inner, or at the lower and inner part of the iris; and the latter when the artificial aperture is to occupy the outer, whether this be upper or lower, part of this membrane.

In performing iridodialysie, we should make an opening into the cornea more or less near its centre; this should be free but not exceeding two millimetres in extent. A small hook is next to be introduced, and the iris laid hold of at its junction with the choroid, and detached from its adhesion. The seized portion is then to be drawn out and fixed between the edges of the wound in the cornea.

To perform iridectomie, we have to make an opening into the anterior chamber more or less laterally according to the situation of the transparent portion of the cornea; next to insert a small forceps, catch hold of a fold of the iris with the blades, draw it out, and excise the projecting portion with fine scissors.

M. *Sichel* assures us that he has seldom failed in obtaining success by adopting either one or other of these modes of operating in almost any case, where the state of the eye in other respects did not preclude the hope of affecting any good.

The application of ocular myotomy, or of the section of one or more of the muscles of the eye, so as to produce a squint, in lieu of forming an artificial pupil, has been approved of by MM. *Cunier*, *Guepin*, Professor *Serre*, &c. We extract the following passage from a letter of the latter gentleman on the subject:........ "Among the various applications of ocular myotomy, there are few, in my opinion, so rational as that which proposes to bring the pupil in relation with a portion of the cornea which retains its transparency. The operation has the great advantage in such a case of being substituted for that of forming an artificial pupil, which must always be one of much difficulty and uncertainty."

*Cataract.*

An elaborate memoir on this subject appears in the *Esculape*, from the pen of Professor *Sichel.* The principal positions, which he endeavours to establish, are these:—

1. That capsular cataract, though infinitely more rare than the lenticular form, is unquestionably of occasional occurrence; its chief diagnostic features being the thickening and elevation of the capsule, and the opacity exhibiting more or less distinct striæ or spots on its surface: the posterior half of the capsule is much less frequently the seat of opacity than the anterior. 2. Capsular cataract is generally the result of inflammatory action; lenticular cataract is rarely so, but is usually induced by age, or by some other causes hitherto unappreciable. 3. The yellowish tint, which the nucleus of the lens so frequently acquires about and after the middle period of life, is quite independent of any opacity, although the hue may increase in depth if a cataract does form. The lens, when it acquires this amber colour, generally looks more or less greenish in the living eye, and is then apt to throw back upon the bottom of the globe a reflected image which may be mistaken for an incipient cataract by the inexperienced oculist.

More than one paper has been recently published on the treatment of cataract without surgical operation; but certainly nothing satisfactory has been made out by many of the writers. M. *Guepin* has been trying the effects of inserting a seton in the temple, not only in the human subject, but also in the horse, an animal which is very subject to cataract.

There seems moreover to be still greater discrepancy of opinion as to the relative advantages of couching and of extracting in cataract. M. *Furnari* says that he believes that the latter operation will ere long be abandoned, except in a few exceptional cases, while M. *Guepin* on the contrary mentions that, as a general rule, extraction is greatly to be preferred to its rival.

It often happens in old persons that, after the extraction of a cataract, the cornea falls in somewhat, and exhibits a depressed in place of a convex surface. However well the operation may have been performed, the patient is unable to see, if this accident takes place. Professor *Maunoir* of Geneva has adopted with success the following expedient to obviate the evil in two or three cases. "Let the patient lie down," says he, "on his back, with his head laid as low as his body, and then let the surgeon pour gently some tepid distilled water over all the exterior part of the orbitar vault, so that the globe of the eye and the lids are completely covered with it. When this is done, let him raise the flap of the cornea (the incision in many cases had been made in the upper half), and the water will enter the anterior chamber of the eye, and fill out the cornea so that it regains its normal convexity, and the patient can distinguish objects before him."

M. *Pirondi* in Italy and M. *Sichel* in France have, during the last two years, been endeavouring to revive the operation of extracting the cataract, by making the wound in the sclerotic coat instead of in the cornea. The latter surgeon advises the incision to be transverse, or in the direction of the fibres of the abductor muscle, and not vertically, as it has usually been made in sclerotomy.

*Amaurosis.*

Several well-written memoirs on this disease have appeared during the course of last year. One of the best is from the pen of M. *Petrequin*, of Lyons, who is favourably known to the profession as a most intelligent and zealous practitioner. There is nothing novel in his observations; nothing that has not been said before; and yet there is a good deal of sound practical instruction to be derived from the perusal of his work. While he agrees with all the best oculists that the cause of amaurosis is very different in different cases—in some being dependent upon an hyperæmia or congested state of the vessels of the retina or choroid coat, in others on an atony or paralysis of the former, and in a third set on sympathy with intestinal derangements—he candidly admits that it is often no easy thing to diagnosticate with accuracy the real cause in individual cases. The remedies, to which he chiefly trusts, are local antiplogistics, the external application of nux vomica—either rubbed on the eyelids and temples, in the form of tincture, or sprinkled upon a blistered surface—belladonna, &c.; and the internal use of mercurials and other intestinal derivatives.

M. *Petrequin* makes a very useful practical remark that ought to be well attended to. In a number of incipient cases of amaurosis, he says, the patient, finding that his sight becomes much shorter than it had been before, at once, and without further ado, has recourse to the use of magnifying glasses. The use of these necessarily aggravates the weakness; and just in proportion as the strength of the glasses is raised, so is the weakness of the vision increased. If, on the contrary, the weak eye was allowed to rest, and was not overstrained, the amaurosis might be in many a case prevented.

M. *Sichel* informs us, as one of the results of his observations, that a good many cases of amaurosis, of apparently the most asthenic character, may be produced or accompanied by a local irritation, or even an inflammatory state, of the retina. He alludes particularly to a form of amaurotic blindness which is occasionally met with in cases of obstinate chlorosis. Along with the internal use of aloetic and steel medicines, it may be necessary to have recourse to cupping, blistering and the local application of strychnine.

M. *Maunoir*, of Geneva, relates a case of amaurosis in which the cure seemed to be effected by the use, for some time, of the following pills:—Extract of arnica ʒij. and sulphate of strychnine 12 grains, to be divided into 144 pills, of which one at first—to be gradually increased to five—was taken night and morning. The latter dose induced muscular twitchings in the back and limbs, like electrical shocks, as well as a good deal of gastric disturbance.

In another case, very decided benefit was derived from dropping into the eye, night and morning, an infusion of capsicum—beginning with three and raising the dose gradually to 30 grains in an ounce of water,—after a host of remedial means had been tried in vain.

Some interesting cases of *hemeralopia*, or nocturnal amaurosis, are recorded in the American Journal of Medical Sciences. In some of them the persons became totally blind at night, and the pupil then was found to be dilated and scarcely to contract when a lighted candle was brought near. A cure was effected by adopting a very simple expedient—viz. giving the eyes entire repose for one, two or three days, by the patients remaining in a darkened room. There had been, no doubt, exhaustion of the nervous energy of the retina, induced by an over-bright light during the day, and it only required rest and sleep, so to speak, to recover its former powers.

MM. *Fleury* and *Frechier* have given a description of an epidemic hemeralopia. The latter gentleman, in the Bulletin de Therapeutique, says: "In the month of March of last year, this strange malady was observed in the district of Maussane. Although pregnant women seemed to be most affected with it, yet no age nor sex was spared.

The degree of blindness differed much in different individuals. In some it amounted to only a weakness of sight coming on after sunset; while others became almost entirely blind as night advanced, although their sight had been perfectly good during the day; in a few instances the eyes continued very weak even during the day."

*Strabismus.*

Every one has heard the French proverb—" there is nothing so new as that which is forgot." Most true this is in a multitude of cases; and not the least remarkably so of many alleged discoveries of modern surgery. It is now found that the operation of dividing one or more of the musles of the eye for the cure of squinting is upwards of a century old.

An itinerant Englishman, of the name of *Taylor*, who called himself oculist to King George the 2nd, published in Paris, as far back as the year 1738, a pamphlet *De verâ causâ Strabismi*, in which he gave an account of his operations.

Eighteen years later, in 1756, a German surgeon, of the name of *Heuerman*, published at Leipsic a work entitled " Abhandlung der neuesten Chirurgische Operationen," or a Treatise on the newest Surgical Operations. In it we find the following passage, which clearly establishes *Taylor*'s claims.

.......... " *Taylor* has also proposed to cure squinting by the division of the tendon of the superior oblique muscle of the eye. But this deformity is not in every case produced by the contraction of this muscle: and moreover the inferior oblique muscle is apt to draw the ball of the eye in the opposite direction when the superior one is divided; thus giving rise to a new sort of squinting. In addition to this, the recti muscles, the contraction of which often occasions squinting, cannot be easily cut across, in consequence of their situation. We thus see that the operation performed by *Taylor* can be only of temporary benefit; and we cannot expect that patients will submit to it, seeing that it is attended with a good deal of pain, and its results are so uncertain."

Well would it be if some of our hasty contemporaries spoke and wrote as rationally as this old German.

M. *Boinet*, in a well-written article in the Journal des Connois. Medico-Chirurg. examines the claims of different surgeons of the present day to the merit of having introduced—or, shall we say, revived?—tenotomy for the cure of strabismus. While he admits that M. *Stromeyer* of Hanover first recommended, and M. *Dieffenbach* of Berlin first executed, at least with success, the operation in question, he argues with much ability in favour of the superior claims of his countryman, M. *Guerin;* as it was he who had previously established the safety and successful results of myotomy and tenotomy in the treatment of various other deformities. This will appear from the following extract from one of his earliest memoirs, the date of which undoubtedly preceded the proposal of M. *Stromeyer*........ " As retraction may affect all the muscles of the economy, we may, by a most logical deduction, extend the operation of tenotomy to every tendon and muscle which occasions any impediment to the normal position of the organs, and instead of limiting it to one or two tendons only, as has hitherto been done, we may lay down a general therapeutic rule for all cases that can occur in practice." Is there not here the *idée-mere*, so to speak, of all that has been done within the last few years in the treatment of strabismus?

The merit of *Stromeyer* and *Dieffenbach* is much about the same as that of the surgeon who may have been the first to apply a ligature to a particular artery for the cure of an aneurism, after the general rules of the operation in such cases had been established by *Anel* and *Hunter*.

There has been a keen dispute between M. *Dieffenbach* and M. *Cunier*, of Brussels, and their friends, as to which of these gentlemen first put M. *Stromeyer*'s proposal into actual practice on the living subject. There is so much discrepancy of statement—proh pudor!—that it is impossible to decide the question. But this is not all; the good faith of some of the Strabotomists—what a word!—has been publicly questioned; they have been accused of wilfully making inaccurate statements as to their success.

A well-written Essay in Latin has recently appeared at Copenhagen, from the pen of M. *Melchior*. In it he states, that having, like other surgeons, been surprised at the alleged marvellously uniform success of *Dieffenbach*'s operations, he went to Berlin with the express object of judging of the truth of the published statements for himself. Of 44 patients, whom he saw operated upon, 10 were perfectly cured, 15 were much benefitted, 9 were triflingly so, and in the remaining 10 the deformity was either quite unaffected or really worse than it had been before the operation. He learned, moreover, that in some of the cases which were successful at first, the squinting returned afterwards; while in other cases the eyeball became so abnormally prominent that one species of deformity seemed only to be substituted for another.

It seems, therefore, that the criticism which M. *Cunier* passed upon the report of *Dieffenbach*'s operations, published by M. *Phillips*, is amply borne out by facts. In that criticism he said—"The reporter is in perfect ecstasies at the brilliant results of the operation. The chapter devoted to the history of ocular contorsion may be summed up in these few words:—A squint being given, divide the muscle on the affected side, and *crack!*.... the eye at once resumes its normal position."

M. *Baudens* has recently discovered a new species of this deformity which, he says, has hitherto never been described!

It must surely be a rare phenomenon; for he tells us that out of 800 cases on which he has operated, he has met with the new species only once. The two eyeballs, he says, are drawn so strongly outwards that two-thirds of the pupil are concealed under the external orbitar angle of the lids; and they cannot by any voluntary effort be brought towards the centre of the orbit. He calls it the *double divergent fixed squint*. M. *Cunier* had previously described it accurately: it belongs to that variety which this experienced oculist has termed *anchylosed*. It is not so rare as M. *Baudens* imagines.

M. *Guerin*, in his memoir read at the Academy of Sciences in January of last year, proposes that all cases of strabismus should be arranged in two groupes. The one form he calls the *mechanical* or the *primitive muscular*, and the other the *optical* or the *consecutive muscular* strabismus. The characters of these two forms are quite distinct; and the treatment required for each is very different. The one is susceptible of being cured, or at least greatly benefitted, by an operation; in the other it is quite inadmissible.

M. *Rognetta*, on the other hand, recognises four kinds of strabismus, according to the supposed cause of the deformity. This, according to him, may be 1, either a congenital or an accidental inequality in the force of the two retinæ; 2, an inequality and want of harmony in the force of the muscles of the eyeball; 3, a mechanical deviation of the visual axis; 4, or a vicious habit derived, perhaps, from imitating persons who do squint.

M. *Cunier*, and subsequently several other writers, have clearly established the fact that not a few cases of strabismus are unquestionably attributable to previous ophthalmia, more especially to the scrophulous and rheumatic varieties of the disease.

### *Various Applications of Ocular Myotomy.*

*Myopia.*—M. *Guerin* was the first to propose the operation in this case. "There are," says he, "two species of myopia as there are two species of strabismus: the one is mechanical or muscular, the other is optic or ocular. The mechanical myopia, like the mechanical strabismus, is the result of the primitive shortness or of the active retraction of the muscles of the eyeball. In M. *Guerin's* opinion, the muscles that are too short, are either all, or two or three at least of, the recti muscles. The contraction must, it will be obvious, be nearly to the same degree in all the affected muscles: otherwise the defect produced would be squinting and not short-sightedness. M. *Cunier* has taken the same view of the question as M. *Guerin*, and he has effected in several cases a cure by dividing the internal and external recti muscles at the same time.

Dr. *Kuh* of Breslaw, in one case, divided all the four recti muscles, and obtained, we are told, "un resultat satisfaisant."(!) This gentleman seems to think that M. *Phillips* of Leige is surely mistaken when he alleges that he has cured myopia by dividing the superior oblique muscle alone; such an operation would, in his opinion, rather aggravate than mend the defect. On the other hand, M. *Bonnet* gives it as his opinion that this imperfection of vision is owing to a contracted state of the inferior oblique, and he consequently recommends the section of this muscle.*

M. *Cunier* has adopted this recommendation in six cases; and what has been the result?—in one case only, the operation was successful; in the other five the shortsightedness seemed to be left worse than it had been before. In four of these unsuccessful cases, he afterwards divided the internal and external recti muscles, and effected a cure of the myopia in three of them; in the fourth case, the operation produced no decided effect. To make amends for this, we find that in one case, where the external and internal recti had been divided without success, the subsequent division of the inferior oblique was followed by a cure!! Well; this is strange work; the poor eyeball is worse treated—to recur to our former illustration—than any ricketty old water-logged coal-brig, whose masts and stays were all leaning over to one side. Would Jack go about nicking with his knife every rope that he found to be rather *taught?* such a bright idea is fit only, we suppose, for the horse-marines.

We quite agree with our brother critic, that "all that is hitherto known as to the indications of myotomy is very incomplete, and that *much* yet remains to be done before we can satisfactorily point out the conditions in which the operation is advisable:"—*very much* indeed.

*Amaurosis.*—It may, on first consideration, appear strange that any one should propose to treat the "drop serene" by dividing any of the muscles of the eye; and M. *Heussu* of Gand is perhaps quite correct in asserting that several of the

---

* So much for the *science* of modern surgery. One man divides two of the recti; another all the four; a third leaves these muscles alone, but he divides the superior oblique; and, lastly, a fourth, in order to make things even we suppose, divides the inferior one.

And yet with such things before our eyes, we are told that surgery has made brilliant advances within the last few years. If she has, it must surely be by one step forward and two backward. There would be no lack, we guess, of materiel for the gibes and jeers of a second *Moliere* in the present day. What with slicing the tongue in a variety of ways for the cure of unfortunate stammerers, and dividing the muscles of the eye for all sorts of visual defects, not to mention the cure of spinal deformities by deliberately cutting across all the contracted muscles of one side of the back, a "pretty considerable tarnation" bit of satire might be worked out, we calculate.

Has it never occurred to any of the bistoury-maniacs of the present day that any painful injury of a weak or irregularly-acting muscular organ may, for a time at least, excite it not only to a more energetic but also to a more normal state of action? and therefore that the apparent cure of stammering, amaurosis, &c. is merely a temporary illusion. Until surgeons have accustomed themselves to watch cases, in which operations have been performed, for a length of time afterwards, we cannot expect a better state of things.—*Rev.*

cases, which have been recorded as instances of success, were in truth cases not of genuine amaurosis, but either of a nervous condition of the muscles themselves, giving rise to a confusion of sight and an incapability of keeping the eye fixed on any object, or to a partial palsy of the third pair of nerves. The following remarks by M. *Petrequin* seems so just as to deserve attention.

"The theory of analogues," says he, "led us to anticipate, and the results of strabotomy have distinctly proved, that the motory apparatus of the eye exercises a marked influence on the functions of its nerves. From what I have observed in several squinting persons, I have been induced to believe that certain cases of amaurosis are primarily owing to a spasmodic or irregularly-acting condition of one or more of the muscles of the eyeball. In strabismus the existence of a muscular spasm is now recognised by surgeons as an acknowledged fact; and it is not the less true that, in the majority of cases of this deformity, there exists a more or less distinctly marked feebleness of vision, more especially on the side on which the deviation is the most considerable. Now I have had occasion to observe that this visual asthenia—whether it be primary or consecutive in its origin is not here the question—generally yields to the section of the contracted muscle or muscles, especially if recourse be had after the operation to due exercise of the eyes (à la gymnastique orthopthalmique.) Myotomy may therefore become a truly heroic remedy for amaurosis arising from this cause; and the important question now is, what are the cases in which it should be performed." M. *Petrequin* says that he has already obtained success in several cases by adopting this line of treatment. As yet he has recorded two of these only.

In one, a youth 18 years of age, there was a partial amaurosis of the left eye, which could not be traced to any of the usual causes of this disease. It was observed, however, that in certain movements of the eye there was a tendency to the ball turning inwards. M. P., on the belief that a spasmodic state of the ocular muscles had a decided influence on the vision, resorted to the section of the two (?) internal recti. The sight of the left eye became immediately improved; the improvement continued; and in the course of five weeks it was quite as good as that of the right.

The other case was very similar; the sight of the left eye had nearly quite gone, without any appreciable cause; but as it was observed that the eyeball had an occasional tendency to being drawn inwards, the operation of myotomy was performed; and an immediate improvement of the sight, we are told, was the result.

M. *Petrequin* has also of late been trying the effects of dividing some of the muscles of the eyeball in what he calls *Kopyopia*—*i. e.* the disposition to fatigue of vision. After stating the results of some operations performed by M. *Bonnet* and himself to shew that this defect of the sight may yield to division of the recti muscles or of the inferior oblique, he proceeds to say, "M. *Bonnet* seems to attribute the complaint to some fault in the oblique muscles, whereas in my opinion it is rather the recti that are at fault: the truth is—and this is the important point—that both the one and the other set of muscles have much to do with the complaint in question." M. *Bonnet* recommends the same operation for kopyopia that he does for myopia—the section of the inferior oblique at its anterior orbitar insertion. For this purpose he introduces, by a puncture previously made in the lower eyelid, a blunt *tenotome*, the point of which he directs backwards and inwards along the line of the floor of the orbit. When it has been passed to the depth of three centimetres, it is then to be carried somewhat forwards so that it may be felt under the skin. The anterior insertion of the inferior oblique may then be hooked and divided.

### *Abnormal Prominence of the Eyeball after Myotomy.*

M. *Cunier* has written more than one paper on this occasional and very unpleasant consequence of dividing one or more of the muscles of the eye. He says:—" The plan recommended for the relief of this condition by MM. *Rognetta, Guerin,* and *Baudens,* consists in attaching, by means of two or three stitches, the inner angle of the lower lid to the corresponding point of the upper one, the operator having first removed with curved scissors a crescentic fold of the integuments. But this operation does not diminish the projection of the eyeball; it only disguises it by inducing a sort of epicanthus. The tension of the eyelids necessarily impedes their free play; and the lachrymal puncta become dragged and compressed, so that the course of the tears is apt to be more or less obstructed. (Such an operation seems certainly a very clumsy and unworkmanlike act to remedy a deformity.—*Rev.*)

What I (M. *Cunier*) propose acts at once on the projecting eyeball, and is moreover not liable to the objections now mentioned. By means of two *crochets-airignes,* I seize a vertical fold between the cicatrix and the caruncula, and excise it with a pair of curved scissors. If the fibrous membrane has not been included in the excised fold, I lift it up with forceps and divide it with one stroke of the scissors. The edges of the wound are then brought and kept together by means of two sutures passed through the conjunctiva and the fibrous membrane. The loss of substance in the conjunctiva and the subjacent membrane induces a shortening and a close adhesion of them with the ball, so that this will no longer retain its abnormal prominence; and at the same time the caruncula, which may have become more or less displaced, regains its natural situation."

M. *Guerin* has not been behind hand in this matter; he also has his plan *de combattre la saillie, la deviation et la perte du mouvement des yeux, consecutives à l' operation du strabisme.* (Is not this a sorry proof of the wonderful cures of squinting that we have heard so much about the last year or two?) M. *Dieffenbach,* too, has been writing on the same subject. His operation, we believe, consists in applying one or two sutures to the conjunctiva of the eye, at the seat of the wound made for the section of the internus muscle.—*Encyclographie des Sciences Medicales.*

---

### SURGICAL TREATMENT OF STAMMERING WELL KNOWN TO THE ANCIENTS.

Poor moderns!—nothing soon will be left to them to make a boast of. Their astonishingly grand discovery of curing squinting in less than a minute by a surgical operation—vaunted as one of the greatest acquisitions of the present century—is discovered to have been perfectly well known a hundred years ago; and alas! too, the kindred operation for the cure of stammering is now shewn, by the very impertinent researches of M. *Joubert,* to have been in common use even before the time of the witty *Rabelais.* Pity it is that this jester of jests did not live in the nineteenth century to record all the marvels that he might have met with in his travels at home and abroad. What a merry tale he might tell of the deeds of the doctors—how a man with a squint of his eye one way went to a surgeon, and how the surgeon performed a wonderful cure; and how the eye then squinted the other way; and how the surgeon again cured this by a second operation; and how the eye afterwards stood out like a lobster's, and how a third time the doctor did something... .............. and how this same patient stammered in his speech, and what the doctor said he would do to cure him of this also; and how he *did for* him at last, &c. &c.

M. *Bourgery,* in giving an account of M. *Joubert*'s historical researches on the subject of stammering, employs this very decided language:—" From these researches it is quite obvious that, under all aspects as to the etiology, the anatomical conditions of the tongue, the operation required, the accidents that may ensue, the chances of relapse, &c. nothing of what we only now begin to know about stammering, was unknown to the ancient authors; the same hopes have induced the same attempts, these attempts being followed by the same disappointments; so that in short the subject, apparently so novel in the present day, has been truly nothing but a mere resuscitation of antiquarian knowledge. It would seem that what one age was perfectly well acquainted with, is like a shut book to the succeeding one; and thus, alas! man moves forwards and backwards, one step now in advance, then a step in a retrograde direction, so that at length he has made just about as much progress in the right road as his forefathers some centuries past."

We shall now briefly mention some examples to prove this point.

*Galen*, in numerous passages of his writings, alludes to various circumstances connected with stammering, especially to the irregularities in the length, thickness, movements, &c. of the tongue itself, which may give rise to this defect of speech. He suggests a variety of means of relief; and among the rest the use of the cautery: but he makes no allusion to any cutting operation.

Nearly four centuries later, *Œtius* has given a very minute description of stammering, the causes that give rise to it, the best mode of relief, &c. The following passage will enable the reader to judge for himself how well acquainted this writer seems to have been with many of the discoveries of modern surgery:

" *De ancyloglossis* et qui vix loqui possunt.*

Of stammerers, some are so from their birth, and others in consequence of disease. In the former, the inferior membranes, by which the tongue is attached, are naturally hard and contracted; while in the latter the incurvation of the tongue in consequence of disease is the effect of a cicatrix from a previous ulceration of the lower surface of the organ.

Persons affected in this manner speak with difficulty, and were thence called by the Greeks Mogilali (*μογος* ærumna, et λαλεω loquor). Those, who stammer from their birth, usually hesitate at first for some time before they commence speaking; but, when the impediment is once overcome, they go on with tolerable volubility: if, however, in the pronunciation of certain syllables, especially when the letters *l* and *k*, or *r* occur frequently, they continue to experience more or less difficulty, a cure cannot be effected without the aid of surgery.

While the tongue is kept up, the membrane, which binds and confines it down, must be divided either with scissors or a scalpel. If an old cicatrix be the cause of the infirmity, this also must be freely divided. The wound should be prevented from uniting, by keeping the edges separated as much as possible until the process of healing is completed."

*Paulus Ægineta* is not less particular in describing stammering:

"The stricture or binding down *(ligatio)* of the tongue, which the Greeks call *ancylo-glossa*, sometimes occurs naturally, and is then owing to the induration and contraction of the membranes which confine it; at other times it results from a cicatrix following an ulceration of the part. In the former case, the person is observed to make every effort in his power to begin speaking, at the same time that the tension under the tongue becomes more obvious. Those, on the contrary, in whom the infirmity has been accidental, will be found to present a cicatrix under the tongue more or less distinct. In the former case—*i. e.* when the complaint is congenital—the operation for its relief is simple: the tongue should be kept well up towards the palate, and then the confining membrane be freely cut across. When, however, it has arisen from the induration and contraction of a cicatrix, something more must be done: the callosity should first be transfixed with a sharp hook, so that the tongue can be drawn well forwards, and then a double lateral incision should be made, so as to free the confinement of the organ, taking care, however, not to make the incisions too deep, as there is considerable risk of a troublesome hæmorrhage supervening."

It is rather a curious circumstance that we do not meet with any notice of impediments of speech again for several centuries after the period of *Paulus Ægineta;* and then the allusion is found in the merry tales of *Rabelais.* His story of the husband, who, having married a wife that could not speak freely, had recourse to a surgeon to cure her, clearly refers to some cutting operation; for we are told: "Le bon mari voulait qu'elle parlat. Elle parla par l'art du medecin et du chirurgien qui lui couparent une *ancyloglotte* qu'elle avait sous la langue."

The good gentleman soon had occasion, we are told, to regret his haste; for his spouse became so loquacious that he returned to the doctor soon after "pour remede de la faire taire."

Less than a century after the time of *Rabelais,*—the beginning of the 15th—we find *Fabricius Hildanus* describing an operation for the cure of stammering and other defects of speech.

It consists in merely snipping the sublingual ligament in several places to give it greater freedom of movement, and in preventing the quick healing of the wounds by moving the tongue about and smearing them with rose-honey, &c.

*Dionis* (1672) alludes to it in the following passage: "we often see children who stammer at the age of four or five years, because the tongue cannot move about with sufficient freedom to enable them to articulate distinctly. To relieve this state of things, we have only to make two or three small cuts with scissors at different points, so as to allow the tongue greater facility of motion."

The author too of the article *Ancyloglossum* in the Dictionnaire Universel de Medecine, published in the course of last century, after citing and commenting upon the writings of most of his predecessors on the subject, gives a minute description of the operation which he recommends. "With the left hand covered with a napkin, or, if this be not sufficient, by means of a particular sort of forceps, the tip of the tongue should be lifted up, and the frænum may then be snipped in several places with a pair of button-pointed scissors. The surgeon may also on some occasions make use of a bistoury, to divide the tissues between the ranular veins and the salivary ducts; but in doing this, great caution is necessary to avoid wounding either set of vessels, as the consequences may be very distressing."

From all these statements it clearly appears that the operation of dividing the sublingual membranes for the relief of certain impediments of speech was quite generally known for several centuries before the commencement of the present; and it now only seems surprising that it should have so entirely fallen into desuetude, until revived within the last year or two—when several of our cotemporaries have actually been disputing among themselves as to their respective claims of its discovery!

However we may disapprove of the method which he recommends, it was unquestionably M. *Dieffenbach* who was the first among them to propose and perform a surgical operation for the cure of stammering, upon its recent resuscitation.

"Before the month of February 1841," says M. *Bourgery,* "no one dreamed of operating upon stammerers, and the public and the profession were engaged solely with the physiological methods of treatment recommended by Madame *Leigh,* MM. *Malbouche, Colombat,* and *Isere.* The latter gentleman did occasionally snip the frænum linguæ, when this membrane appeared to be unusually tight. It was on the 1st of April that the Journal des Debats announced M. *Dieffenbach's* first operation in these words. 'This distinguished surgeon has found out a method of curing stammering by making an incision in the tongue: the operation has succeeded perfectly. His opinion is that stammering is attributable to the confinement of the tongue so that it cannot be applied to the palate; the operation consists in correcting this state of things.' This announcement created a great sensation among the surgeons of Paris. M. *Dieffenbach* having not at this time made public his method of operating, every one immediately set himself to devise one for the purpose. If we can believe M. *Phillips*, who has made himself the historian of this period, he operated upon two patients on the 6th of February, and next day he addressed a letter to the Academy. He it was, we believe, who had the good sense first to transfer the cutting operation from the upper or dorsal to the inferior or sublingual part of the tongue. On the 14th of the month the cases of MM. *Velpeau* and *Amussat* occurred; and in the course of this and the following months many other surgeons engaged in the same enquiry. From this period the surgical operation for the cure of stammering *put commencer à entrer dans l'enseignement.* (Indeed; we very much doubt it.) It seems now very generally admitted that *Dieffenbach's* method is utterly inadmissable, and ought decidedly to be expunged from surgery. If anything is ever to be attempted with the knife, it must be in the sublingual region—thus shewing, as often happens in medicine, that we unwittingly return to the forgotten acts of our predecessors, while we fondly imagine that we have been making some original discovery."—*Gazette Medicale.*

### Obliquity of the Nose cured by Operation.

M. *Dieffenbach*—who seems determined that no deformity of limb or body should perversely resist the magic of the knife—has been trying of late his orthopædic skill on two young men, whose noses had got awry, and had become turned to one side. In one of the cases the obliquity of the nasal member was congenital; in the other the calamity had been caused by a fall upon this awkwardly jutting-out promontory of the human face divine. In both of the unfortunates the deformity, we are candidly informed, was "des plus choquantes;" the nose no longer, as in duty bound, appeared to be in the middle of the face; but it was completely thrown outwards and lay all *couchant* on the cheek, one nostril being directed upwards and the other downwards.

A small bistoury was slipped under the skin of the side of the ala nasi, just at the place where the cartilage joins the bone of the nose, and it was then turned so as to divide the point of union of the nose with the bones on each side—all this, be it remembered, was done *more subcutaneo,* the integuments remaining unwounded except where the bistoury, was introduced. The "falling tower" of Naso, being thus loosened at its foundations, could easily be propped up erect; and all that there was then necessary to do was to secure it in its new and honorable position. The surgical architect, we are told, was eminently successful in every step of the proceeding; and so satisfactory were the results that no one could suspect that the building had ever been in fault at all.—*Caspar's Wochenschrift.*

### M. Stromeyer on Spasm of the Thumb in Writing.

The celebrated Hanoverian surgeon M. *Stromeyer,* Professor of Surgery in the University of Erlangen, has published the reports of two cases of this very troublesome complaint, in which he tried the effect of dividing certain muscles of the affected thumb.

Before narrating these, we wish to allude to a few circumstances connected with what has been called by some "the writers' cramp," as it would seem that more than one morbid state of the digital muscles or tendons is included under this appellation. In the Medico-Chirurgical Review for April 1840, will be found a short notice of several cases, from one of the German Journals, of spasmodic tremor of the fingers in writing, the tremor in some instances extending up the fore-arm and arm, and accompanied with a greater or less degree of pain. In M. *Stromeyer's* cases it seems that the thumb was almost exclusively affected; while in those related by Professor *Langenbeck* of Gœttingen, in a recent number of the Allgemaine Zeitung fur Chirurgie, &c. the affection appears to have consisted chiefly in a spasmodic contraction of one or more of the external muscles of the forefinger. In one case, he divided the common and proper extensors of this finger, immediately below the point where the former muscle sends off a communicating slip to the tendon of the middle finger; but the result was any thing but fortunate; and we understand that in two other similar cases still worse consequences ensued. We shall find that M. *Stromeyer* was more fortunate, at least in one of the following cases.

*Case* 1.—A gentleman, 32 years of age and of a delicate constitution, had for two years been afflicted with what has been called the *writers' cramp* (spasmus habitualis flexoris pollicis longi). It had become so distressing that he was quite disabled from writing with his right hand; all that he could do was merely to scribble a few almost illegible words; and often before he could do even this, a convulsive movement of the thumb came on, jerking the pen fairly from between his fingers and scattering the ink about. And yet, strange to say, he could use his right hand for doing any thing else without difficulty or inconvenience. While the patient was writing, it was obvious that the muscles of the thenar eminence were unusually rigid—a circumstance which naturally suggested the idea that these muscles were the seat of spasm. As every thing that could be thought of had already been ineffectually tried to relieve this most distressing affection, M. *Stromeyer* determined to divide the small muscles of the thumb, avoiding any injury to the tendon of the long flexor. The operation had certainly no good effect; for the spasm continued as bad as ever, and to this there was unfortunately added a loss of sensation in the palmar face of the thumb. M. *Stromeyer* was now convinced that the action of writing was dependent upon the long flexor of the thumb, and he wished to divide it; but the patient refused to submit.

*Case* 2.—A schoolmaster, 45 years old, robust and plethoric, has for nearly fifteen years been affected with the *writers' cramp,* which prevents him not only from writing but also from playing on musical instruments. When he attempts to write, he is obliged to steady the thumb of the right hand with the thumb and forefinger of his left one, and, even with this assistance, he cannot do more than write a few lines at most. When he touches the notes of a piano, the thumb is immediately drawn in towards the palm of the hand, and the second phalanx is powerfully flexed. And yet this patient can use his right hand in all other manual exercises without trouble or unusual fatigue. During the efforts to write, there is no apparent rigidity of the small muscles of the thenar eminence, as was observed in the preceding case. This circumstance, added to the *brusque* flexion of the second phalanx in playing upon the piano, induced M. *Stromeyer* to try the section of the long flexor muscle of the affected thumb;—although this muscle did not seem to be at all contracted, nor to impede in any way the free movements of the thumb. The deep seat of the muscle made the operation somewhat difficult to divide it alone and without injury to any other parts. The patient being made to bend as strongly as he could the first phalanx of the thumb, at the same time that it was forcibly drawn outwards, M. *Stromeyer* inserted, about the middle of this phalanx, a narrow curved *tenotome* at the side of the tendon, down to the bone; the point of the instrument was passed under the tendon, and this was divided at a single stroke by moving the hand "en bascule." When the instrument was withdrawn, the sensibility of the thumb was found to be much impaired; the dorsal surface recovered it on the first, and the palmar surface on the fourth day after the operation: upon the latter day the power also of moving the second phalanx returned. On the 15th day the patient made an attempt to write and to play on the piano: he did not experience the slightest cramp, and found that he could do both acts with the greatest ease.

Experience alone can determine whether the simple section of the long flexor of the thumb will be found sufficient for the cure of this most distressing complaint, or whether it will not be sometimes necessary to divide any other muscles likewise.

M. *Stromeyer* dwells at considerable length on the influence which division of the muscles of a limb exercises upon its sensibility. This influence is the more decided in proportion as the muscles are more powerful and active. Hence it is usually very marked in cases of accidental rupture of the tendo-achillis in robust persons; the limb becomes almost quite insensible, and this loss of sensation continues more or less until the two ends of the divided tendon become united. The return of sensibility is usually indicated by a feeling of pricking or creeping in the part. When the muscles to be divided are considerably wasted and have lost much of their energy, the loss of sensation is generally much less considerable. These phenomena are especially remarkable after the section of the digital tendons: the loss of sensibility is usually very decided: it is sometimes however limited to the palmar surface when the flexors alone are divided. The sensibility usually returns—but in most cases not till then—with the returning motility of the member. It is very questionable whether this loss of sensation is dependent upon the division of any nervous filaments; if it were so, would it cease so soon as we observe it to do? Genuine paralysis from section of a nerve does not cease so rapidly. It is certainly not easy to explain the circumstances; and it may be wise therefore at present to state the fact, without endeavouring to explain it.—*Bayerisches Med. Corresp. Blatt.*

*Remarks.*—Although we readily acknowledge that it is no easy thing to suggest a remedy for the peculiar spasmodic affection of the fingers alluded to in the preceding remarks, we should feel very unwilling to recommend a cutting operation, with the view of dividing any of the tendons or muscles which seem to be chiefly affected, except indeed in an extreme case, where the mischief was confined exclusively to one tendon, and provided also every other rational means of relief had been found ineffectual. Does it not seem that the convulsive action of the fingers in the *writers' cramp* is nearly of the same nature as the irregular and truly convulsive movements of the tongue in most cases of stammering?—perhaps indeed the appellation of *stammering of the fingers* expresses the nature of the complaint in question better than any other. The muscles of both parts are thrown into vibratory and—if we may coin a word—*dis-sentaneous* contractions in the attempt to perform certain voluntary actions, and in these only; and thus, instead of the harmonious co-operation of all in one direction, certain muscles become affected with violent spasm, while the others are more or less quiescent. All know that most stammerers can articulate their words with sufficient distinctness and facility in singing, and also that the impediment of speech is greatly less while reciting a passage from a foreign language than when conversing in their mother tongue. Whenever in short the person is obliged to perform the acts of inspiration and expiration with greater regularity than usual, and especially if his attention is withdrawn at the same time from the remembrance of his infirmity, he will find that his stammering is infinitely less than under opposite circumstances. Well, and so it is in a great measure with the peculiar affection of the thumb and fingers, which some persons experience in writing. There is nothing positively altered or diseased in the state of any of the digital muscles or tendons; far from it; the spasm, with which they are apt to be seized, is only occasional, and moreover it may be made to cease in a moment, by merely discontinuing the attempt to write. In some cases, the person continues able to use the hand with the greatest dexterity in playing upon a musical instrument or in any other delicate work; it is only when the pen is taken into the hand that the cramp or spasm comes on. But whether this be the case or not, no difficulty or distress is experienced in using the hand in ruder and rougher occupations—as for example in boxing, rowing, dressing and so forth. Now have we not seen that the case of the stammerer is very much alike—he can sing, nay often can recite long passages from Latin, or any other language whose syllables are deep-toned and sonorous, without difficulty; and yet, when he attempts to speak a few words in his mother-tongue, he is at once seized with the most distressing spasms. The two cases are therefore very strikingly parallel; and the treatment therefore of both should surely be managed on somewhat similar principles—viz. by withdrawing, as much as possible, the attention from the affected part, and by disciplining it to perform slow, and regularly-repeated movements. In both cases—although as a matter of course the precept is of much more importance to him who stammers with his tongue than to him who stammers with his fingers—the patient should be directed to avoid all hasty movements; and one of the best rules of all with this view, is to regulate the breathing, so that the acts of inspirations and expirations are even and well sustained. Whenever the breathing is performed, so to speak, slovenly—we mean, now deep and long, now short and hurried, now at regular and then at irregular intervals—every person, however robust he may be, will find that he is more or less nervous, easily thrown off his guard, and apt to be more than usually excited or depressed.

To allude at greater length here to the treatment of stammering would be entirely out of place. Suffice it to say, that no surgical operation is necessary in one out of a hundred cases. If there be a manifest shortness or rigidity of the frænum, we should never hesitate to snip it across, that the tongue might have greater freedom of movement; but even this is very rarely necessary. The infirmity may generally be got rid of, or at least very greatly relieved, by proper discipline of the voice and breathing; without slicing the tongue as M. *Dieffenbach* recommends, or snipping off the tonsils, as Mr. *Yearsley* is, or was, in the habit of doing with such marvellous success!

By-the-bye, this scientific gentleman should be on his guard, or else he will soon gain for himself the reputation of a thorough quack. We recently saw a youth on whom he had operated without the slightest benefit, but who was subsequently cured of his stammering in the course of two or three days by Mr. *Hunt,* whom we have heard favourably spoken of on several occasions, and who, we are glad to observe, is patronised by several distinguished members of the profession.

But to return to the subject of spasmodic affections of the fingers, we may again remind our readers that they will find some illustrative cases, with remarks, in the number of the *Medico-Chirurgical Review* for April 1840. We there (p. 519,) expressed our opinion that we knew of no plan to relieve the distress but that of ceasing to use the finger at the time when the affection came on and of diverting the mind so as to withdraw the attention from the seat of suffering. We recently received an interesting letter from Mr. *Miller,* of Kilmarnoch, in which he alludes to a case of this description that had occurred in his practice. He says:—"I have a patient who has for a year or two complained very similarly to *Giere's* (the second case referred to in your Journal) and one about whom I have considerable anxiety, as he is besides a highly valued personal friend. The affection, too, threatens sometimes to incapacitate him almost entirely for his profession, that of an attorney. He is of a very keen sensitive temperament, and I rather think that electioneering excitement and annoyances gave rise to the complaint. For a considerable time at first his stomach was greatly at fault, and I was disposed to blame this for the other affection: but the stomach and whole body have now got into much better tone, and the annoying tremor of the fingers in writing continues unabated. As general remedial means, his diet has been regulated, he has been put upon tonics of various kinds, has taken out-door exercise, used the cold bath, flesh-brush, &c.; while locally all manner of anodyne liniments have been tried."

Mr. *Miller* kindly asked us if we could suggest any means likely to remove this troublesome complaint. If we remember aright, we advised that the chief reliance must be on brisk manual exercise, such as the use of the dumb-bells, pitching a weight, pulling an oar, &c. and the cessation of writing whenever the tremor comes on—the mind being all the while kept as tranquil as possible from fidgetty fears as to the consequences, and the general health being attended to. The more anxious that the patient is in such cases, the more obstinate is the complaint likely to prove; if he can be induced to make light of it, the chances are that some morning it will take wings to itself, and flee away.

Before dismissing the subject of digital spasm, we may mention that we recently saw a well-marked case of severe spasm of the tendon of the extensor longus of the great toe. The woman was a middle-aged woman, subject to plethora of the head. The spasm of the muscle was often for several days so great that this toe was forcibly drawn up to nearly a right angle with the others, so as quite to prevent her wearing a shoe on the foot. Neither internal nor external means had any effect: the tendon was therefore divided; and the relief thus afforded has now continued for several months.—(*Rev.*)

---

### Report of Vaccination in France during 1840.

M. *Claubry* communicated to the Academy of Medicine, at the meeting held on the 26th of April last, the conclusions of the Annual Report of the Vaccination Commission of France for the year 1840. From this report it appears that the number of vaccinations performed in the different departments of France during this year has been 525,509, while the number of births was 836,789.

In two cases, which have been made known, there was a general eruption of vesicles over the body; and the lymph, taken from these vesicles, was found to communicate on inoculation a regular vaccine pock.

The epidemic variola affected 14,470 persons; of these 1,668 died, and 1,390 remained more or less disfigured and enfeebled. There were 24 instances of second attacks of variola; in three of these the disease proved fatal.

An immense majority of the vaccinated persons escaped entirely the influence of variola. Some were affected with a varioloid affection or a sort of modified variola, which was usually very mild and occasionally resembled the vaccinia. Of 406 vaccinated persons, who were affected in different degrees of severity, six only died.

Of 2,214 re-vaccinations, there were 1704 cases in which it failed; 227 in which a pseudo-pock was formed; and 270 in which perfectly normal vesicles appeared. Three re-vaccinated persons only were subsequently affected with a varioloid affection.

The following conclusions are appended to the general report:—

1. The vaccinia protects the human system from variola. This protection is however not indefinite in every case; for a certain number of vaccinated persons still remain subject to an eruption which is known by the name of the varioloid.

2. This eruption, although of a variolous nature, is usually mild and unattended with danger. During the year 1840, the proportion of deaths from this disease was only about one per cent.

3. A first vaccination destroys the aptitude for a second one, as it does the susceptibility to the attacks of variola. There are, however, some persons in whom this aptitude is reproduced after the lapse of a certain period, and there are likewise persons who have been affected with variola, in whom the vaccinia is developed in a regular form, without our being able to determine whether they were liable to contract a second attack of variola.

4. The most complete success of re-vaccination is not to be considered as an absolute protection against the chances of an ulterior attack of variola.

5. In general, variola attacks a person only once. There are, however, some persons who are not exempt from a second attack of the disease, and this second attack has been known to prove more severe that the first.

From the following remarks made on the preceding conclusions of the report by different members of the Academy, the reader will be enabled to judge somewhat of the prevailing opinions in France on the subject of vaccination, &c.

M. *Moreau* wished that the report should state distinctly that vaccinated persons are not preserved from variola more than those who have already had the variola once.

M. *Double* asked the reporter if he considered that the occurrence of the varioloid in an individual authorises us to infer that he has not been protected from the variola. According to his (M. *Double's*) opinion, the varioloid and the genuine variola are two diseases which are quite distinct from each other. *Rhazes* was the first to express this opinion; and subsequently *Van Swieten*, and, in our own days, M. *Rayer* have adopted it.

M. *Honoré* seemed to take the same view of the case as M. *Double*. He alluded to the mistake—which, by-the-bye, is by no means uncommon—of supposing that the varioloid is merely a form of the variola modified by previous vaccination. So far from this being the case, he said, it is abundantly obvious from the writings of last century, that the varioloid was perfectly well recognised long before the introduction of vaccination; and indeed it is well known that the very difference of opinion among the older physicians, as to genuine variola ever occurring twice in the same individual, arose in a great measure from the circumstance that many authors viewed the varioloid as a disease which was quite distinct from the variola.

M. *Piorry*, on the other hand, was inclined to regard the two diseases as the same, the varioloid being merely a mild form of the variola. In all epidemics, he said, we observe cases in which the genuine variola seems to be transmitted by persons affected with the varioloid only, and *vice versâ*. It is said by some that the variola is always much more dangerous. True, but the danger of the disease is attributable almost exclusively to the extension of the eruption to the larynx and trachea. (This unqualified assertion contains a grievous error. The danger of variola is owing much more to a vitiated state of the fluids of the body than to any local affection whatsoever. It is necessary on all occasions to check the localising pathology of so many of the French writers.)

M. *Barthelemy* repeated the question already put by M. *Double*, and enquired of the reporter whether it distinctly followed from the documents, which had been sent to the Academy, that genuine variola is really apt to be sometimes communicated by persons affected with the varioloid. To this question M. *Claubry* answered distinctly in the affirmative.

M. *Rayer* gave it as his opinion that the two diseases, variola and the varioloid, are owing to the same contagion—the matter of the contagion only acting in one case with more intensity than in another, or else meeting with certain individuals less predisposed than others to be affected with it.

In the course of all epidemics we have often occasion to observe that the varioloid makes its appearance in persons who already have had the variola, or who have been vaccinated or inoculated; but it is also developed in persons who are not placed in any of these circumstances, and who have not had either the cow-pox, or the natural or acquired small-pox.

M. *Velpeau* stated that he had met with several cases which to his mind seemed distinctly to prove that the two diseases were really the same in nature, although each had its particular shades of difference. He recently met with a case in point which occurred in a young gentleman of his acquaintance, who had been vaccinated in his infancy: after visiting a friend labouring under variola, he fell sick with it, and the eruption proved confluent; but, at the period of suppuration of the pustules, they all flattened and, so to speak, withered away, without any purulent formation. One of the cousins of the patient caught a mild varioloid from him; and then this last patient's mother, who had already had the small-pox, became affected with the varioloid.

M. *Bousquet*—who has paid great attention to all the questions touching the history of small-pox and cow-pox—expressed his most decided conviction that variola and varioloid are essentially the same disease. Not only do the two eruptions usually appear together at the same time and in the same places, but they evidently spring the one from the other. During the three last years, a number of small-pox and varioloid patients have been admitted into the Hôtel Dieu under the care of M. *Chomel,* who took especial pains to ascertain the origin of the disease in each instance; and he ascertained in almost every one that the vaccinated persons, who were affected, had been exposed to contact with variolous patients. He also has satisfied himself that the varioloid communicates variola with all its attendant dangers to those who have not been protected by vaccination.

Direct experiments have led to nearly the same results. M. *Lafont* of Toulouse, so far back as 1818, and subsequently some other physicians, have very clearly shewn that genuine variola may be communicated to an unvaccinated person by inoculation with the matter of varioloid pocks. As an additional proof that the two diseases are essentially the same, it deserves to be mentioned that an attack of the varioloid appears to afford as much, or nearly as much, protection against variola, as a previous one of variola itself. Acting upon this principle, M. *Guillon* in 1826—from being unable to obtain a supply of vaccine lymph during the prevalence of a very fatal epidemic of small-pox in Finistere—tried the effects of inoculating upwards of 600 persons with the matter taken from a varioloid eruption with which a vaccinated person happened to be affected. By singular good luck, the disease so generated had in almost every instance the characters of varioloid and not of genuine variola. M. *Bousquet* adds—but with what correctness it is not easy to say—that none of these 600 persons have been subsequently affected with variola.

"If," continued he, "I thus dissent from M. *Double* in one respect, I quite coincide with his opinion upon another—viz. that the varioloid was well known long before the discovery of vaccination, and indeed that its origin is probably coeval with that of its sister variola."

In the writings of the physicians of last and of the preceding centuries, we meet with frequent allusions to cases of difficulty, which some regarded as instances of small-pox, and others as examples of chicken-pox. The distinction by former writers of the small-pox into that which is of *long* and that which is of *short* duration, seems to correspond with the distinction now made between genuine variola and the varioloid.

With respect to the third conclusion or proposition of the report—that which has reference to the effects of re-vaccination—the following discussion took place.

M. *Moreau* said that, in his opinion, it was impossible to determine with accuracy the period at which a second vaccination will produce decided effects. He had met with the most discordant results on this point in his own practice. He stated that in his youth he had had the small-pox, and that subsequently he has been vaccinated nearly a dozen times; and that upon three different occasions the vaccination succeeded perfectly.

M. *Bousquet* was greatly inclined to doubt the accuracy of M. *Moreau*'s opinion, even in reference to the effects which he said had been three times produced by vaccination in his own person. That the vaccine virus when inserted under the skin may give rise to very decided local effects repeatedly, may be quite true; but it does not follow from this that there is a re-production each time of the normal cow-pox vesicle: far from it. The experience of M. *Bousquet* has quite satisfied him that re-vaccination soon after the first operation is usually followed by only an abortive attempt to form the regular pock. It will be acknowledged to be still a very difficult matter to determine, how long the first vaccination protects the system against the contagion of small-pox, and when it usually begins to lose this preservative power.

That those, who have had small-pox, may be quite susceptible of the influence of vaccination was well known to *Jenner*; for he has distinctly stated that 'the cow-pox protects the system from small-pox; but there is not a reciprocity of action.'

It must be confessed that we are not warranted in the belief that the susceptibility of the system to a second vaccination is, *ipso facto*, indicative of its susceptibility to the contagion of small-pox: this subject requires much more examination than it has hitherto received, before we can arrive at any decided conclusions on the subject.

---

### OXYDES OF IRON AS COUNTER-POISONS TO ARSENIC.

M. *Guibert* was, we believe, one of the first to determine with care how far the moist precipitated oxyde of iron might be trusted to as an antidote to arsenic. His inquiries were, on the whole, decidedly favourable to it; and the accuracy of his statements has been confirmed by the unexceptionable testimony of MM. *Miguel*, *Soubeiran*, and others. Mr. *D. Maclagan*, of Edinburgh, has recently published a paper with a view of shewing that the oxyde precipitated from a solution of the sulphate by ammonia is considerably more efficient as an antidote than the same dose of the oxyde precipitated by caustic potash. M. *Guibert*, after a series of carefully-performed experiments, admits the correctness of Mr. *Maclagan*'s assertion; the general conclusions to which he has come are these:—

That five parts of the oxyde of iron, in the state of moist hydrate,—whether this has been obtained by using caustic potash or ammonia—precipitate *entirely* one part of arsenious acid from its solution. If four parts only of the oxyde be used, then it will be found that the precipitation of the arsenic is still complete with the ammoniacal oxyde, but not so with that prepared with potash. It is to be observed that much larger proportions of the oxydes of iron than those now mentioned must be exhibited with the view of counteracting the effects of poisoning with arsenic in a living animal—the above remarks being applicable only when the oxyde is allowed to remain in contact with the arsenical preparation for 24 or 48 hours.

MM. *Guibert* and *Maclagan* both agree that the hydrated oxyde of iron in the dry state is very much inferior as an antidote to the moist *magma*. The common subcarbonate, kept in chemists' shops, is still weaker than the dried hydrate. M. *Guibert* found that it "is at least three times less powerful in neutralizing arsenious acid than the dried hydrate, and six times at least less powerful than the hydrate in a moist state." This, however, clearly shews that the common subcarbonate, or sesqui-oxyde as it is now called, is not altogether powerless as an antidote, and therefore that if the hydrate is not at hand, we should at once give large and repeated doses of it diffused in water, until the hydrate can be prepared.—*Bulletin de Therapeutique.*

